# Diversity and history of the long-chain acyl-CoA synthetase (*Acsl*) gene family in vertebrates

**DOI:** 10.1186/1471-2148-13-271

**Published:** 2013-12-12

**Authors:** Mónica Lopes-Marques, Isabel Cunha, Maria Armanda Reis-Henriques, Miguel M Santos, L Filipe C Castro

**Affiliations:** 1 CIIMAR – Interdisciplinary Centre of Marine and Environmental Research, CIMAR Associate Laboratory, UPorto, University of Porto, Porto, Portugal; 2 ICBAS (Instituto de Ciências Biomédicas Abel Salazar), University of Porto, Porto, Portugal; 3 Department of Biology, Faculty of Sciences, University of Porto, Porto, Portugal

**Keywords:** acyl-CoA long chain synthetase, Gene loss, Genome duplication, Differential paralogue retention, *Acsl2*

## Abstract

**Background:**

Fatty acids, a considerable fraction of lipid molecules, participate in fundamental physiological processes. They undergo activation into their corresponding CoA esters for oxidation or esterification into complex lipids (e.g. triglycerides, phospholipids and cholesterol esters), a process that is carried out by acyl-CoA synthases (ACS). Here we analyze the evolution of the gene family encoding for the long-chain acyl-CoA synthetases (*Acsl*) in vertebrates.

**Results:**

By means of phylogenetics and comparative genomics we show that genome duplications (2R) generated the diversity of *Acsl* genes in extant vertebrate lineages. In the vertebrate ancestor two separate genes originated the current *Acsl*1/5/6 and the *Acsl*3/4 gene families, and the extra gene duplicates in teleosts are a consequence of the teleost specific third round of genome duplication (3R). Moreover, the diversity of *Acsl* family members is broader than anticipated. Our strategy uncovered a novel uncharacterized *Acsl-*like gene found in teleosts, spotted gar, coelacanth and possibly lamprey, which we designate *Acsl2*. The detailed analysis of the *Acsl2* teleost gene *locus* strongly supports the conclusion that it corresponds to a retained 2R paralogue, lost in tetrapods.

**Conclusions:**

We provide here the first evolutionary analysis of the *Acsl* gene family in vertebrates, showing the specific contribution of 2R/3R to the diversity of this gene family. We find also that the division of ACSL enzymes into two groups predates at least the emergence of deuterostomes. Our study indicates that genome duplications significantly contributed to the elaboration of fatty acid activation metabolism in vertebrates.

## Background

Two rounds of genome duplication (1R and 2R) have now been clearly established to have occurred in early vertebrate evolution [1], with a further round taking place in teleost ancestry (3R) [2]. Extra independent genome duplications have been determined in salmonids [3], and in ray-finned fish paddlefish [4]. These events have modeled the genomes of extant vertebrate lineages in terms of gene numbers and the overall genome architecture, contributing to the appearance of numerous innovations [5]. In addition to the increase in gene counts resulting from 1R/2R/3R, the comprehension and detection of gene loss processes in combination with the differential retention of paralogues poses important challenges to enlighten vertebrate evolution [6-9].

Fatty acids (FA) are a particularly important category of lipid molecules, being a considerable source of energy and a significant component of bio-membranes. FA metabolism involves among others, processes such as hydrolysis, beta-oxidation, synthesis, esterification and activation. The later comprises a two-step, ATP dependent reaction, with the formation of an intermediate acyl-AMP which is then converted to acyl-CoA. Acyl-CoA synthetases (ACSs) are the key enzymes responsible for this fundamental initial step in lipid metabolism. They can act on non-polar hydrophobic FA substrates and convert them into water-soluble products (acyl-CoAs), which are then integrated into metabolic pathways such as oxidation of acyl-CoAs to obtain ATP, storage in the form of triglycerides (TGA) or use as building blocks for other lipid molecules. The human genome contains 26 ACS genes divided into 6 distinct families: Short-chain ACS family (ACSS); Medium-chain ACS family (ACSM); Long-chain ACS family (ACSL); Very long-chain ACS family (ACSVL), Bubblegum ACS family (ACSBG) and ACS-Family (ACSF) [10-12]. This division reflects the chain length of their preferred substrate. ACSL enzymes play a paramount role in humans, since FAs with 12 to 20 carbons (C12-C20) are highly prevalent in the diet and are preferentially converted to acyl-CoA by these enzymes [12,13]. Further, several pathological conditions have been linked to ACSL enzymes such as inadequate lipid metabolism leading to diabetes [14], X-linked 63 mental retardation (MRX63-OMIM300387) [15,16] and cancer [17]. In mammals, previous studies identified five distinct *Acsl* genes, which were further organized into two separate groups (*Acsl1*, *Acsl5* and *Acsl6*) and (*Acsl3* and *Acsl4*) [10,12,18]. It is worth mentioning that the current *Acsl* gene nomenclature omits the “*Acsl2”* which was dropped since shortly after its identification it was found to be identical to *Acsl1* in human and additionally rodent “*Acsl2*” was also renamed *Acsl6* since it shared more identity with human *Acsl6*[18]. The advent of whole genome sequencing projects allowed the identification of *Acsl* genes in non-mammalian species, but no approach has been made in order to unravel the evolutionary history of this family [12,19]. Additionally, recent findings have illustrated the need to consider genome duplication processes (and gene loss) in combination with extensive species analysis for proper evolutionary insights regarding lipid metabolic gene networks to be drawn [20,21]. Moreover, non-mammalian species, such as the zebrafish, have been recently proposed as alternative models to study lipid metabolism [22]. Therefore, a comparative and phylogenetic approach involving a broader number of vertebrate species should shed light into the evolutionary history of *ACSL* enzymes and their function. In this study we demonstrate that genome duplications in stem vertebrate ancestry and the teleost specific genome duplication were instrumental in the generation of *Acsl* gene diversity. Moreover, we show that the variety of *Acsl* family members is broader than anticipated. Our strategy uncovered a novel uncharacterized *Acsl-*like gene found in teleosts and coelacanth, which we designate *Acsl2*. The detailed analysis of the *Acsl2* teleost gene *locus* strongly supports the suggestion that it corresponds to a retained paralogue, lost in other vertebrates classes (“*an ohnolog gone missing*”). Finally, we provide the first comparative transcription analysis between the human and zebrafish *Acsl* gene repertoire.

## Results

### ACSL gene repertoire in vertebrates

Human ACSL1, ACSL3, ACSL4, ACSL5 and ACSL6 sequences were used to perform Blastp searches and collect *Acsl*-like sequences from various available genomes. We analyzed a total of 21 species in order to include all major vertebrate lineages. Our database search determined the presence of five *ACSL* genes in humans, mouse, opossum, chicken, anole lizard, western clawed frog, and the coelacanth. In the spotted gar, an out-group of the teleost specific genome duplication [23], we found 5 sequences though 2 were partial (Additional file 1). Blast searches in teleost fish genomes hinted at a larger *Acsl* gene set, with nine hits in zebrafish, pufferfish, green spotted puffer and medaka and seven in stickleback. However, detailed sequence analysis suggested a number of inconsistent annotations in the Ensembl database. For example, we found three *Acsl1* gene annotations in medaka, (1-ENSORLG00000019563, 2-ENSORLG00000018806 and 3-ENSORLG00000008655), however, when aligning the DNA and amino acid sequence of the first two sequences we observe that they are identical (not shown). Given that the annotated *Acsl1* copy ENSORLG00000018806 is located within a contig that presents extensive regions that are poorly resolved we consider that this species presents 2 gene copies of *Acsl1* and select ENSORLG00000019563 and ENSORLG00000008655 for further studies. The green spotted pufferfish again shows three annotated copies of *Acsl1* with two of these (ENSTNIG00000000345 and ENSTNIG00000010115) located in the same scaffold with the same orientation and contiguously (Additional file 2). These annotations are partial sequences, one corresponding to the N-terminal and the other corresponding to the C-terminal of the protein. Here we assume that these annotations correspond to a single gene poorly assembled. Therefore we consider that the green spotted puffer presents two *Acsl1* genes and we use only the correctly annotated gene (ENSTNIG00000018054) for further analysis. Finally we find two annotated *Acsl1* genes in pufferfish (1-ENSTRUG00000017576 and 2-ENSTRUG00000001450) were the second gene corresponds to a partial sequence which was not used for further analysis. We investigated also the genomes of three Chondrichthyans, the elephant shark, catshark, and little skate. Our investigation identified 4 full sequences and several partial (Additional file 1).

Finally, the search in the lamprey genome resulted in four *Acsl-*like gene hits (1-ENSPMAG00000008135, 2-ENSPMAG00000004625, 3-ENSPMAG00000005099 and 4-ENSPMAG00000005133). Three of these correspond to partial sequences (449 residues) and were not used for further analysis. Finally, in the investigated invertebrate species, acorn worm and amphioxus, we recovered 3 *Acsl* sequences from acorn worm and 4 *Acsl* sequences from amphioxus. After clarifying all inconsistent gene annotations a set of ACSL sequences from various species were collected to perform phylogenetics (Additional file 3).

### Phylogenetics indicates vertebrate specific Acsl gene expansions

**Figure 1:**
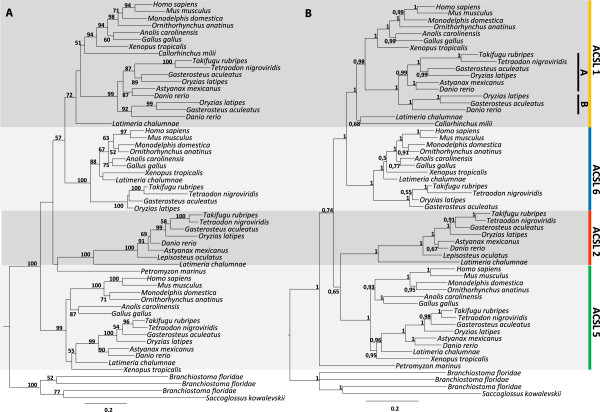
**Phylogenetic analysis of Acsl1, Acsl2, Acsl5, Acsl6 sequences. (A)** Maximum likelihood tree, numbers at nodes represent percentage of bootstrap values (only values above 50% are shown); **(B)** Bayesian tree, number at the branch nodes refer to posterior probabilities.

**Figure 2:**
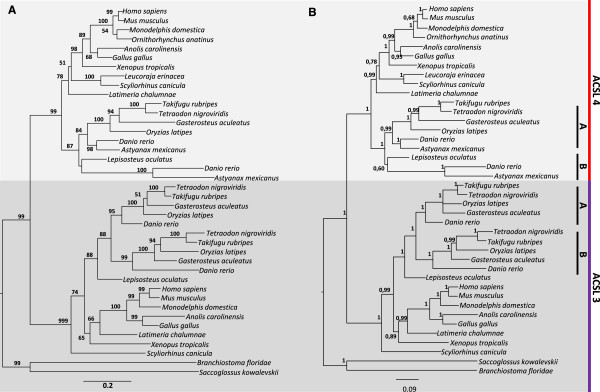
**Phylogenetic analysis of Acsl3 and Acsl4 sequences. (A)** Maximum likelihood tree, numbers at nodes represent percentage bootstrap values (only values above 50% are shown); **(B)** Bayesian tree, number at branch nodes refer to posterior probabilities.

Preliminary phylogenetic analysis confirmed that *Acsl3* and *Acsl4* form a distinct group from *Acsl1*, *Acsl5* and *Acsl6* as previously reported (data not shown) [10,12,18]. Thus, we have reconstructed each phylogeny separately (Figure 1 and Figure 2). In the Maximum likelihood analysis Figure 1A it is possible to observe that invertebrate sequences out-group four statistical well supported clades comprising *Acsl1*, *Acsl5*, *Acsl6* and an unidentified *Acsl* group. However, the exact phylogenetic relationships between each isoform are not statistically supported with the bootstrap analysis. In the Bayesian analysis (Figure 1B) we find again the invertebrate sequences out-grouping four statistically well supported vertebrate clades. The unidentified *Acsl* group is composed of teleost, rayfin fish and coelacanth sequences. In the Maximum likelihood analysis a lamprey sequence also groups with this novel clade (though weakly supported). We name this new gene lineage *Acsl2*. The overall tree branching pattern in Maximum likelihood and Bayesian analysis is indicative that the expansion of *Acsl1*/*5/6/novel* clade took place after the radiation of the vertebrate lineage approximately 500 million years ago, although independent gene expansions have taken place in amphioxus and the acorn worm (Figure 1A and B). We find representatives of *Acsl1/5/6* in all of the examined vertebrate species, with the exception of lamprey and chondrichthyans where the presence of partial sequences impedes a final conclusion regarding the full *Acsl* gene repertoire in these lineages (see Additional file 1). Nevertheless, this cannot be taken as an indication of gene loss due to the poor genome sequence coverage. The phylogenetic trees also indicate that *Acsl1* has specifically duplicated in the teleost lineage. Even though only medaka, zebrafish and stickleback present these duplicates, we antecipate that pufferfish and the green spotted pufferfish probably retain these two copies.

Regarding the *Acsl3* and *Acsl4* trees (Figure 2), both in the Maximum likelihood and Bayesian analysis we observe that the invertebrate *Acsl-*like sequences again out-group two well supported groups containing vertebrates sequences. Also, it is possible to recognize that all teleost species here analyzed present a lineage specific duplication of *Acsl3* (*Acsl3a* and *Acsl3b*). In zebrafish and cave fish we find an *Acsl4* duplicate; microsynteny analysis of this *locus* in zebrafish suggests that this extra gene copy is also a teleost specific 3R duplicate (Additional file 4). However, despite extensive database search, we did not retrieve other *Acsl4-*like sequences in other teleost species. *Acsl3* and *Acsl4* gene copies were also found in the cat shark and little skate (Figure 2).

The phylogenetic analysis also resolves a further inaccurate annotation in the western clawed frog. In the Ensembl database two ORFs are annotated as *Acsl4* genes (1-NP_001090679.1-ENSXETG00000033126 and 2-ENSXETG00000012429). After observing the localization of these two sequences in the phylogenetic tree, we find that one of the annotated “*Acsl4”* groups within the *Acsl3* clade, which is consistent with a synteny analysis. Therefore we consider that the western clawed frog presents one *Acsl4* gene (ENSXETG00000012429) and one inaccurate annotation of an *Acsl3* gene (ENSXETG00000033126) (Additional file 5).

In summary, the phylogenetic data indicates that the *Acsl1*, *Acsl2*, *Acsl5* and *Acsl6* and *Acsl3* and *Acsl4* have all duplicated before vertebrate radiation, with episodes of lineage specific expansion observed in studied invertebrate deuterostomes*.* In addition, teleost fish underwent specific duplications in *Acsl1* and *Acsl3*, and possibly *Acsl4* in zebrafish and in cave fish.

### Genome duplications contributed to the diversity of *Acsl* genes in vertebrates

**Figure 3:**
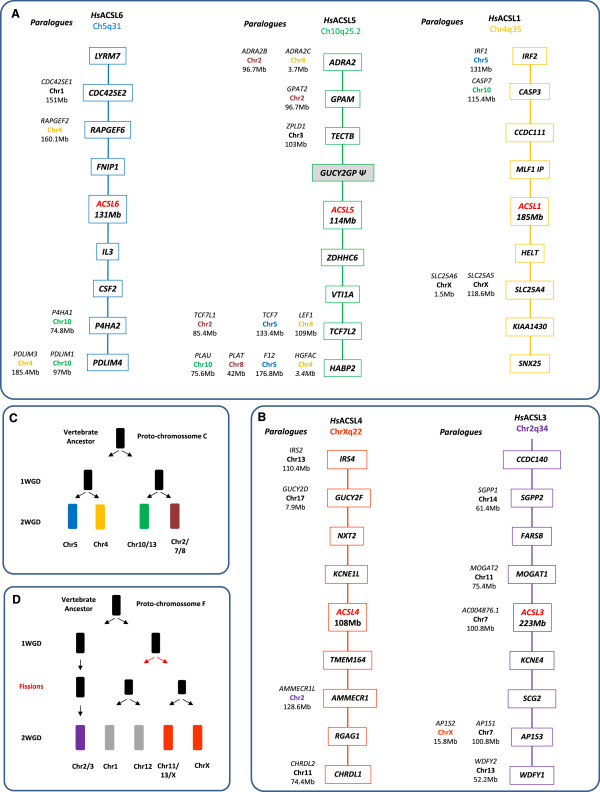
**Microsyteny analysis of the *Acsl *human *loci *and their mapping location in the ancestral vertebrate chromosomes. (A)** Location of the *Acsl1, Acsl5, Acsl6* and neighboring genes in the human genome and corresponding paralogues; **(B)** location of *Acsl3, Acsl4* and neighboring genes in the human genome and corresponding paralogues; **(C and D)** schematic representation of the duplication history of the ancestral vertebrate chromosomes C and F.

Our phylogenetic analysis clearly indicates that despite the existence of several *Acsl* gene copies in the studied invertebrate deuterostome species, the expansion of the *Acsl1/Acsl2/Acsl5*/*Acsl6* and of the *Acsl3*/*Acsl4* clades took place in the vertebrate ancestor. Thus, we next analyzed the contribution of 2R and 3R in the generation of *Acsl* gene diversity. We started by examining the genomic location of each human *ACSL* gene and respective flanking gene families (Figure 3). Human *ACSL1, ACSL5 and ACSL6* localize respectively to Chr4q35, Ch10q25.2 and Ch5q31 (Figure 3A), regions which are part of the 2R NK-paralogon [24-26]. The analysis of the flanking gene families revealed that those which are multi-copy and whose duplication timing coincides with vertebrate emergence, typically have their members localizing to Hsa4, Hsa5, Hsa8, Hsa10 and/or Hsa2. For example, *TCF7L2* gene which flanks *ACSL5* has two other duplicates mapping to Hsa4 (*LEF1*) and Hsa5 (*TCF7*); *CASP3* which maps close to *ACSL1* has a paralogue, *CASP7*, mapping close to *ACSL5*; *PDLIM4* mapping downstream of *ACSL6* has two paralogues, *PDLIM1* and *PDLIM3*, localizing to Hsa10 and Hsa4 respectively. Overall, the majority of genes flanking human *ACSL1*, *ACSL5* and *ACSL6* revealed conserved macrosynteny and therefore support the hypothesis that these regions are related, with the duplication timing coinciding with 2R. Furthermore, using the proposed vertebrate ancestral genome reconstruction [27], we find that the Hsa4, Hsa10 and Hsa5 belong to the same ancestral group, group C (Figure 3C). In summary, from a single ancestral chromosome C in the vertebrate ancestor, derived four chromosomes (C0, C1, C2 and C3) [27] as a result of 1R/2R. Each human *ACSL locus* maps to a distinct ancestral C chromosome: *ACSL1*-C1, *ACSL6*-C0 and *ACSL5*-C2 (Figure 3A and C). We would expect to find a fourth *ACSL* gene which should map to the ancestral chromosome C3 distributed in present day human genome at Hsa2/7/8 (see next section).

Regarding human *ACSL3* and *ACSL4* genes we find that they map to chromosomes Chr2q34 and ChrXq22 respectively. Neighboring gene families have paralogues in the following set of chromosomes HsaX, Hsa2, Hsa7, Hsa11, Hsa13, Hsa14 and Hsa17 (Figure 3B), with no apparent conserved macrosynteny. However, *ACSL3* and *ACSL4* map to chromosome regions derived from the 2R duplication of the proto-chromosome F, at F0 and F4 respectively (Figure 3D). Accordingly, after the first round of genome duplication one F proto-chromosome underwent an additional fission event, resulting in three proto-chromosomes. Two of these proto-chromosomes underwent the second WGD, giving rise to four ancestral chromosomes (F1, F2, F3 and F4) and the third chromosome gave rise to the F0. The gene families flanking *ACSL3*/*ACSL4* have in most cases duplicates in regions assigned to F chromosomes [27], thus providing strong support to the hypothesis that both were 2R generated.

**Figure 4:**
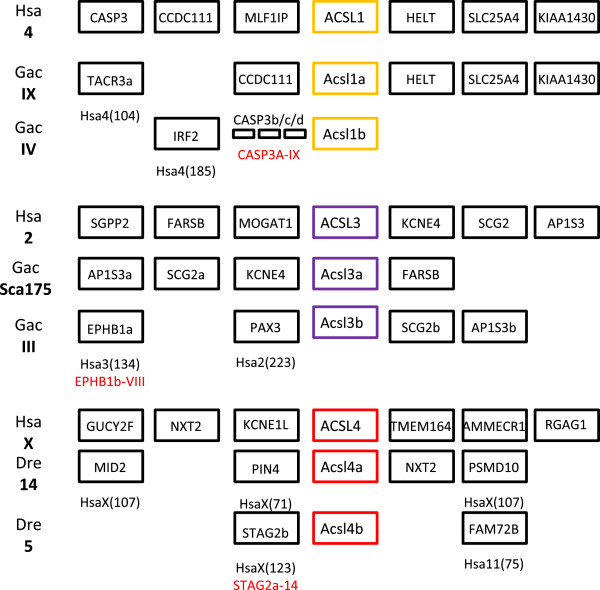
**Conserved synteny of *Acsl1*, *Acsl3 *** **and *Acsl4 *** **between human and teleost (*G. aculeatus *** **and *D. rerio*) indicate the contribution of 3R in the generation of extra gene copies.**

Extra gene copies of *Acsl1*, *Acsl3*, and *Acsl4* (in zebrafish) were found in our survey. The analysis of the *loci* of *Acsl1*, *Acsl3* and *Acsl4* (Figure 4) in stickleback and zebrafish provides solid support to the conclusion that 3R contributed for the increase of the *Acsl* gene set in teleosts. We find that 3R specific duplicates can be observed outflanking each pair of *Acsl* duplicates (*Casp3*, *Ephb1* and *Stag2*) (Figure 4).

### *Acsl2* is a potential 2R paralogue gone missing in tetrapods

**Figure 5:**
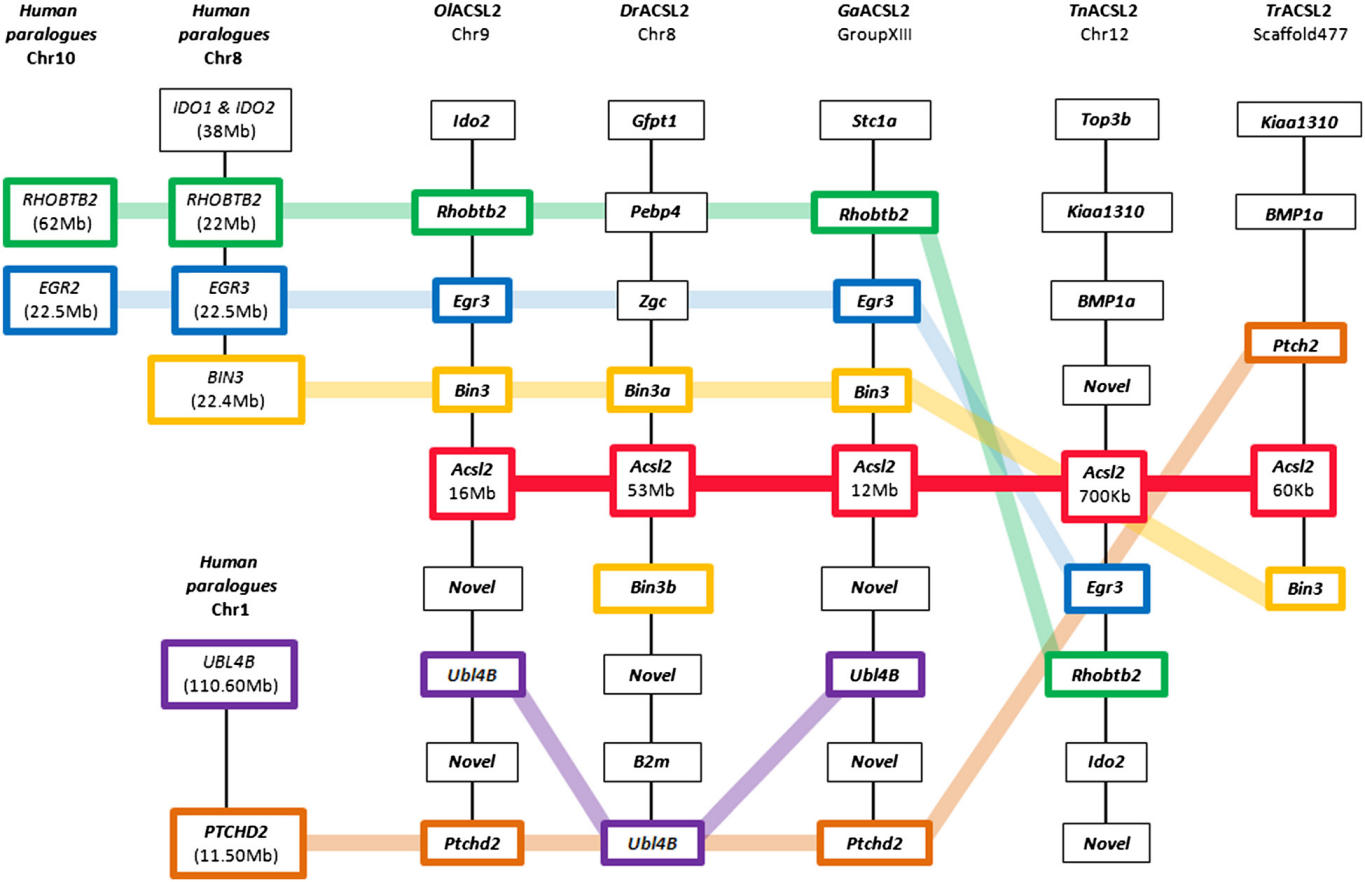
**Synteny map of *Acsl2 loci *** **in teleost fish, and the location of human orthologues.**

The phylogenetic analysis showed the presence of a previously unidentified *Acsl* gene, *Acsl2*, paralogous to *Acsl1, Acsl5* and *Acsl6*. To enlighten the evolutionary origin of the *Acsl2* gene we analyzed the genomic *locus* of this novel gene in teleost species (Figure 5). We show that the *Acsl2* gene is confined to a largely conserved *locus* in teleost fish. A large set of neighboring gene families have their human orthologues mapping to Hsa8. The following genes *EGR3, BIN3, RHOBTB2*, *BMP1*, *PEBP4*, *STC1*, and *IDO2* are close together at Hsa8 and constitute a conserved block, with *UBL4b* and *PTCHD2* localizing in Hsa1 (Figure 5; data not shown). Most importantly, the conserved syntenic block in Hsa8 maps back to the ancestral chromosome C3 which corresponds to the expected localization of a fourth copy of the *ACSL* gene after 2R, absent in the human genome. Further, gene families which are multicopy have their paralogues localizing to Hsa10/5/4 as expected. These finds together with the phylogenetic analysis suggest that the *Acsl2* gene corresponds to a retained paralogue conserved in teleosts and lost in the tetrapod lineage.

### Gene expression data indicates functional partitioning and diversification

**Figure 6:**
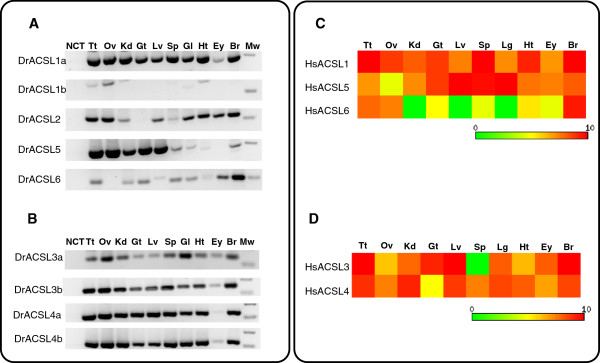
**Comparative expression analysis of *Acsl *genes in human and zebrafish tissues.(A and B)** Tissue expression profile of zebrafish *Acsl* genes; **(C and D)** Tissue expression heatmap of Human *Acsl* genes; NCT negative template control, Tt: testis, Ov: ovary, Kd: kidney, Gt: gut, Lv: Liver, Sp: spleen, Gl: gill, Ht: heart, Ey: eye; Br: brain, Lg: lung, Mw: molecular weight marker.

Given that the teleosts have additional *Acsl* gene copies, we proceeded to analyze the gene expression of *Acsl* genes in zebrafish and performed a comparative analysis with the human *ACSL* gene repertoire. In zebrafish a high *Acsl1a* mRNA content is observed in all analyzed tissues with the exception of the eye (Figure 6A), while *Acsl1b* is only marginally expressed in testis, ovary, kidney and heart (Figure 6A). The human *ACSL1* has a similar expression pattern with high expression in brain, heart, spleen, kidney, ovary and testis and medium to low expression in liver, lung and eye (Figure 5C). These findings are in agreement with previous studies in *Rattus norvegicus* in which it was found that *Acsl1* is highly expressed in major energy metabolizing tissues namely heart, liver and adipose tissues [28]. Regarding ACSL5, in human we find that this enzyme is highly expressed in all tissues here analyzed with the exception of the ovary (Figure 6C). When observing the data obtained for zebrafish we find a distinct expression pattern. High *Acsl5* mRNA transcription is observed in the testis, ovary, kidney, gut and liver, while spleen, gill, heart, eye and brain have a low/absent gene transcription (Figure 6A).

Concerning ACSL6, in opposition to ACSL1 and ACSL5; this enzyme presents a fairly restricted expression pattern in human being highly expressed in testis, ovary and brain. In zebrafish this restricted expression pattern is also observed, with *Acsl6* being found essentially in brain (Figure 6A). The teleost *Acsl2* transcription is high in testis, ovary, gill, heart, eye, and brain.

The expression pattern of *Acsl3a* and *Acsl3b* in zebrafish, reveals that *Acsl3a* is preferably expressed in ovary, gill and brain; nevertheless this gene is also expressed at lower levels in all other tissues (Figure 6B). *Acsl3b* is expressed in all tissues with comparatively higher levels to *Acsl3a,* with the exception of eye. The expression pattern of the *Acsl4a* and *Acsl4b* is highly similar being highly expressed in all tissues here analyzed with a slight decrease in gut and liver and low expression in eye (Figure 6B). When observing the expression pattern of *ACSL4* in human we observe that this gene is also highly expressed in all tissues with the exception of gut and eye (Figure 6D).

## Discussion

**Figure 7:**
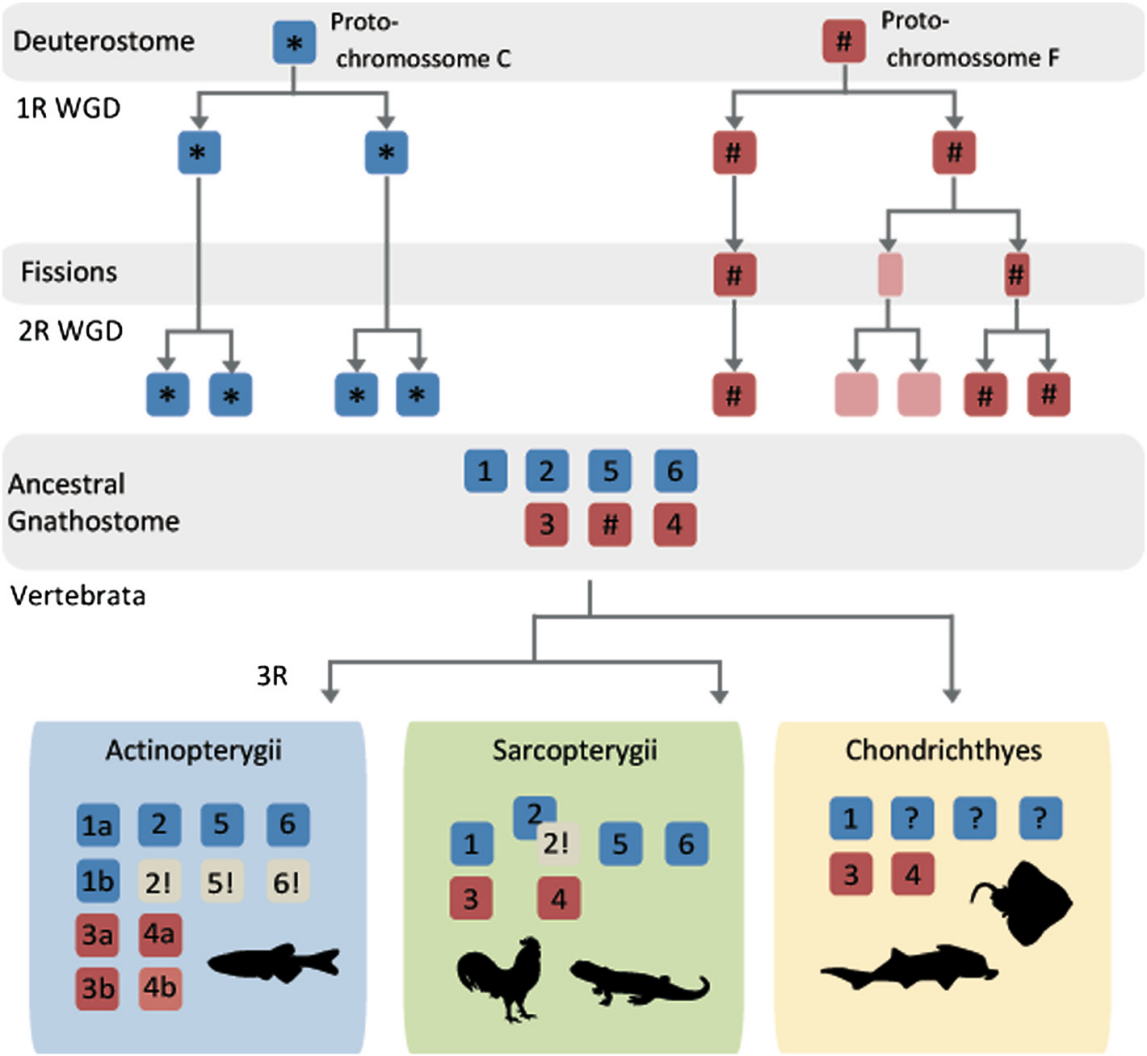
**Proposed evolutionary history and duplication timing of the *Acsl *gene family in vertebrates.** Questions marks indicates unknown data, and exclamation signals gene loss.

ACSL are key enzymes involved in the initial steps of FA metabolism. These enzymes preferentially activate FA with C12-C20 (the most abundant in the human diet), which may then intervene in a variety of metabolic pathways. Although the *ACSL* family has been the focus of various studies, their evolutionary history has not been investigated before. Here we combine extensive database search with phylogenetics and comparative genomics to provide a reliable depiction of the evolution of *Acsl* in vertebrates (Figure 7). Initial analyses revealed several inaccurate gene annotations. After an exhaustive analysis we were able to clarify several of these and perceive that the diversity of *Acsl* genes is broader than anticipated. The gene repertoire varies significantly between mammals (5), teleosts (7/8), and the invertebrate studied species (3/4). Through phylogenetics we were able to reconstruct the *Acsl* gene duplication timings in relation to the divergence of major vertebrate groups. The five mammalian *Acsl* genes have been organized into two separate groups: *Acsl1*, *Acsl5*, *Acsl6* and *Acsl3*, *Acsl4*, on the basis of sequence homology and gene organization [10,12,18]. We now propose that this division is evolutionarily significant and dates back to at least deuterostome ancestry since clear co-orthologues of both gene groups exist in hemichordates and cephalochordates. Although, various *Acsl1/2/5/6* genes were found in amphioxus and the acorn worm, these represent independent lineage duplications. The exact duplication timing of a proto-*Acsl* gene to originate the ancestor of *Acsl1/2/5/6* and *Acsl3/4* is at present unknown but probably dates as far back as the origin of the Bilateria (not shown; LFCC unpublished results). In the agnathan lamprey we were only able to retrieve one complete *Acsl* sequence, although several partial sequences were also evident. Thus, at this stage a final conclusion concerning the full repertoire of *Acsl* genes in lamprey is premature. The findings that the *Acsl* gene family expanded significantly in the time window coincident with the emergence of vertebrates lead us to test the contribution of genome duplications. Using the proposed ancestral vertebrate genome reconstruction [27] we were able to trace back the duplication history of *Acsl* genes in the gnathostome ancestor. We find that human *Acsl1, Acsl5 and Acsl6*, map to chromosomes C1, C2 and C0 respectively which originated from duplication of the ancestral proto-chromosome C (Figure 7). This observation is also supported by the duplication history analysis of the flanking gene families. In teleosts we found extra gene copies within the *Acsl1/5/6* clade. These were partially explained by the contribution of the teleost specific genome duplication (3R) (*Acsl1a* and *Acsl1b*), but not entirely. A novel gene with no clear orthologues in tetrapods was found in the analyzed teleost species, the spotted gar, coelacanth and possibly lamprey. The phylogenetic analysis clearly indicated that this represents a distinct gene lineage which we name *Acsl2*. To enlighten the origin of *Acsl2*, we investigated the genomic *locus* in teleosts and cross-compared it with the human genome. We find that the most parsimonious explanation for the retrieved data is that *Acsl2* is a 2R paralogue retained in teleosts and lost in tetrapods, similar to what was found in a distinct ACS gene family [21]. Thus, the novel uncharacterized *Acsl2* gene corresponds to a quadruplicate *Acsl* paraloguous to *Acsl1, Acsl5* and *Acsl6*.

Similarly, we found also that the human orthologues of *Acsl3* and *Acsl4* map to chromosome regions related by duplication. Both genes map to genomic regions remnants of 2R resulting from the duplication of the ancestral proto-chromosome F. The most plausible explanation for the unequal distribution of *Acsl* gene copies within F0, F1, F2, F3 and F4, is a chromosome fission event occurred after 1R of WGD which resulted in the splitting of the genetic information into two distinct chromosomes, which then underwent the second genome duplication (2R) and originated F1, F2, F3 and F4 (Figure 7). The detailed comparative genomic and phylogenetic analysis again highlighted the contribution of 3R to the gene number increment observed in teleosts (Figure 7).

It has been previously suggested that major vertebrate innovations occurred after genome duplication events [5]. Whole genome duplications lead to the expansion of gene numbers, facilitating gene diversification, sub-functionalization along with the rise of novel functions and gene loss. These ultimately enable evolutionary radiation. FA composition and metabolism is known to be different in some vertebrate groups, for example in teleosts [20]. Similar to our findings in *Acsl*, recent studies have also revealed that various gene families involved in lipid metabolic pathways have evolved distinct gene repertoires in vertebrate lineages, including fish, with clear functional and regulatory impacts [20,21,29,30]. The retention of such a larger *Acsl* gene set after 2R/3R, with simultaneous processes of differential loss, could be indicative that novel *Acsl* gene functions have emerged in vertebrate ancestry. In effect, the variety of *ACSL*s in mammals is apparently associated with distinct substrate preferences. ACSL1 uses FA with C16 to C18 both saturated and mono unsaturated, ACSL3 displays a high activity with C12:0 (laurate), C14:0 (myristatate), C20:4 (arachidonate) and C20:5 (eicosapentaenoic acid) [31]. In contrast, ACSL4 presents a clear preference for polyunsaturated FA with C2O:4 and C20:5 [10,32]. ACSL5 shows substrate specificity similar to ACSL1, favorably utilizing palmitic (C16:0), palmitoleic (C16:1), oleic (C18:1) and linoleic (C18:2) acids [33]. Finally, ACSL6 preferentially uses FAs with C16 to C20 saturated and polyunsaturated, although alternative splicing generates isoforms with distinct substrate specificities [10,34]. We propose that teleost orthologues have probably retained similar FA substrate preferences with respect to *Acsl*s. However, novel *Acsl* family members were also discovered in this study. Thus, we performed a comparative tissue transcription analysis between zebrafish and human. We selected zebrafish as a model, given that this species has been previously suggested as a model organism for the study of lipid metabolism [22], and coincidentally this species also presents the largest set of *Acsl* genes. The comparison of the expression data between human and zebrafish revealed a similar expression profile, with the exception of *Acsl2* and *Acsl5,* and the fish specific duplicates (*Acsl3* and *Acsl4*)*.* We observe that the zebrafish *Acsl5* is expressed in fewer tissues when compared to the human orthologue, with the teleost *Acsl2* apparently compensating the lack of *Acsl5* transcription in various tissues. This setting suggests that in teleost fish functions are shared between *Acsl2* and *Acsl5*. Regarding the 3R duplicated members *Acsl3a* and *Acsl3b*, and *Acsl4a* and *Acsl4b* we find similar tissue expression distributions in opposition to *Acsl1a* and *Acsl1b*. In the latter *Acsl1b* is co-expressed with *Acsl1a* in a small set of specific tissues (testis, ovary and heart). We hypothesize that *Acsl1b* plays a specific role in these tissues, distinct from the role played by *Acsl1a*, hinting towards a sub-functionalization after duplication. Although, we have not tested the FA specificity of the novel *Acsl* repertoire described in this work, we cannot ignore that the retention of a larger *Acsl* gene number in teleosts could also be related with the specific acquisition of novel substrate preferences, which future studies should address.

## Conclusion

In summary, we demonstrate the importance of genome duplications, 2R and 3R, in the generation of the Acsl diversity in vertebrate species.

## Methods

### Database mining and identification of Acsl sequences

ACSL family members were identified in the Ensembl, GenBank and JGI (Joint genome institute) databases through Blastp searches using as reference annotated human ACSL sequences. In order to include all major vertebrate lineages we analysed eutherian metatherian and prototherian mammals: *Homo sapiens* (human), *Mus musculus* (mouse); *Monodelphis domestica* (opossum); *Ornithorhynchus anatinus* (platypus); birds: *Gallus gallus* (chicken); reptiles: *Anolis carolinensis* (anole lizard); amphibians: *Xenopus tropicalis* (western clawed frog); Latimeria chalumnae (Coelacanth); *Lepisosteus oculatus* (spotted gar); teleosts: *Danio rerio* (zebrafish), *Astyanax mexicanus* (blind cave fish), *Takifugu rubripes* (pufferfish), *Tetraodon nigroviridis* (green spotted puffer) *Oryzias latipes* (medaka) and *Gasterosteus aculeatus* (stickleback); chondrichthyans: *Leucoraja erinacea* (little skate), *Scyliorhinus canicula* (small-spotted catshark) and *Callorhinchus milii* elephant shark*,*and jawless fish hyperoartia: *Petromyzon marinus* (sea lamprey). Sequences searches were also made in an invertebrate chordate *Branchiostoma floridae* (amphioxus) and the hemichordate *Saccoglossus kowalevskii* (acorn worm).

### Sequence alignment and phylogenetic analysis

All ACSL amino acid sequences retrieved in the database mining were initially aligned in MAFFT alignment software using default parameters [35] and manually curated with the exclusion of regions of uncertain homology, gaps, and of partial sequences. Revised sequence alignments were then submitted to Protest online server version 2.4 [36] available at http://darwin.uvigo.es/software/prottest_server.html, in order to select the appropriate protein evolution model according to our dataset. Here we found that *ACSL3* and *ACSL4* group follows a JTT + I + G model, while the LG + I + G + F model suits best the *ACSL1*, *ACSL2 ACSL5* and *ACSL6* group. Phylogenetic analyses were performed and a Maximum likelihood (PhyML) tree with 1000 bootstrap replicates was constructed using the online platform of the PhyML 3.0 avaliable at http://www.atgc-montpellier.fr/phyml/. Bayesian inference of phylogeney was performed with MrBayes version 3.2.2 [37] on CIPRES Science Gateway [38]. Analysis for both Acsl3/4 and Acsl1/2/5/6 amino acid sequences were performed under a mixed substitution model, with two parallel runs with 1 million generations, each with four chains one cooled and 3 heated. Trees were sampled every 100 generations; final consensus tree was calculated with the fifty percent majority rule and from the remaining trees after a 0.25 burin.

### Comparative genomics

All *ACSL* genes were mapped into the human chromosomes, the location of each gene and the neighboring genes were collected from Ensembl and GenBank databases. *ACSL loci* in human were used as a model for comparison. The Ensembl paralogue and orthologue prediction tools were used to infer duplication history patterns of flanking ACSL genes. For some flanking gene families we run phylogenetics to confirm relationships with the methodology described above (not shown).

### Gene transcription analysis

Adult wild-type zebrafish obtained from our own breeding stock were used for gene expression analysis. Animals were anesthetized and killed by cervical transection in accordance with the Portuguese Animals and Welfare Law (Decreto-Lei nº 197/96) approved by the Portuguese Parliament in 1996. Institutional animal approval by CIIMAR/UP and DGV (Ministry of Agriculture) was granted for this study. After collection all tissues were preserved in RNAlater and stored at -20°C. Total RNA was isolated using an Illustra RNAspin Mini RNA Isolation Kit (GE Healthcare, UK) according to the manufactures recommendations, including the on-column treatment of isolated RNA with RNase-free DNase I. RNA concentration was calculated using Qubit fluorometer instrument (Invitrogen, Carlsbad CA), integrity confirmed by electrophoresis and the RNA stored at -80°C until further use. The cDNA was synthesized from 250 ng of total RNA with the iScript cDNA Synthesis Kit (Bio-Rad) according to the manufactures protocol. Forward and reverse primers were designed using sequences available in Ensembl with Primer3 software [39]. All of primer sets match exon sequence and flank an intron consequently avoiding genomic DNA amplification. Primers sets were created for the following genes *ACSL1a*; (Forward-5′ CAGGATGGGCAAAGAATAGAG 3′, Reverse-5′ TTTCAGTGTTGGTGTGAGGAG 3′, annealing at 55°C) *ACSL1b;* (Forward-5′ GCACAGCGAGATGTTCAC 3′, Reverse-5′ AAGTCCAATCCAAATGTCAGG 3′, annealing at 54°C) *ASCL2* gene; (Forward-5′ GTAGTTCCAGATCCAGAAGTGTTC 3′ reverse-5′ CGCCGTCATGTCCTCCAG 3′, annealing at 56°C) ACSL5; (Forward-5′ CGCAGAGAAACTGGGATTGAAAGG 3′, Reverse-5′ TGGCTTTGAGTGTTGGAGTGAGG 3′, annealing at 58°C) and *ASCL6* (Forward-5′ CCTCGTGGGCTCAGAAGAAAG 3′ Reverse-5′CGCACCATGTCCTCCAGAATA 3′, annealing at 58°C). PCR was performed using 2 μl of zebrafish cDNA and Phusion® Flash high-fidelity Master Mix (FINNZYMES). PCR parameters were as follows: initial denaturation at 98°C for 10 s, followed by 35 cycles of denaturation at 98°C for 1 s, annealing for 5 s and elongation at 72°C for 10s and a final step of elongation at 72°C for 1 min. PCR products were then loaded onto 2% agarose gel stained with GelRed and run in TBE buffer at 80 V. *In silico* expression analysis, for *ACSL* gene in Human, was performed using ESTs available from Unigene [40] as count per million transcripts, all values are displayed as Log2 transcripts per million. Heat map was created using the collected EST data and matrix2png web interface v1.2 [41].

## Competing interests

The authors declare that they have no competing interests.

## Authors’ contributions

The original idea for this study was conceived by LFCC. LFCC and MLM performed all the experimental analysis; MMS, MARH, and IC participated in the discussion regarding lipid metabolism and physiology. The manuscript was written by LFCC and MLM, and edited by all other co-authors. All authors have read and approved the final manuscript.

## Supplementary Material

Additional file 1 tBlastn search of ACSLlike sequences in Transcriptomic contigs. Click here for file

Additional file 2 **Partial** ***Acsl*** **gene annotations in green spotted pufferfish.** Click here for file

Additional file 3 NCBI accession numbers and Ensembl gene ID. Click here for file

Additional file 4 Synteny maps of Zebrafish ACSL 3R duplicates. Click here for file

Additional file 5 **Xenopus tropicalis *Acsl4 *** **and *Acsl3 *** **corresponding location in human.** Click here for file
