# Supplementary material for: Diversity and history of the long-chain acyl-CoA synthetase (Acsl) gene family in vertebrates
Source: BMC Evol Biol. 2013 Dec 12;13:271. doi: 10.1186/1471-2148-13-271 (PMC3890633; doi:10.1186/1471-2148-13-271)
Supplement: Additional file 1 — tBlastn search of ACSLlike sequences in Transcriptomic contigs. [file 1471-2148-13-271-S1.pdf]

Additional file 1

tBlastn search of ACSLlike sequences in Transcriptomic contigs

tBlastn searches were performed on Transcriptome Contigs in elephant shark, little skate, small spotted catshark (http://skatebase.org) using as reference human ACSL protein sequences, contigs containing hits were retrieved. Ensembl database was used to retrieve annotated ACSL genes in Lamprey and Coelacanth while Pré-Ensembl database was used for Spotted Gar.

Sequence sorting and profiling was done using phylogenetic analysis with partial sequences and Blastp in NCBI

	Acsl1/Acsl2/Acsl5/ Acsl6	Acsl3/Acsl4
Elephant shark	ctg60368 (f)ACSL1<br>ctg48116 (p) ACSL6?	ctg17226 (p)ACSL3?<br>ctg79717 (p)ACSL3?<br><br>ctg24956(p)ACSL4?<br>ctg26023 (p)ACSL4?<br>ctg18935(p)ACSL4?<br>ctg34934 (p)ACSL4?
Little Skate	ctg11347 (p) ACSL1?<br>ctg17167 (p) ACSL1?<br>ctg82046 (p) ACSL2?<br>ctg34321 (p) ACSL5?<br>ctg43339 (p) ACSL5?<br>ctg31550 (p) ACSL5?	ctg12475 (p)ACSL3?<br>ctg14318 (f) ACSL4
Cat shark	ctg18625 (p) ACSL1?<br>ctg94905 (p) ACSL1?<br>ctg63419 (p)ACSL2?<br>ctg81762 (p)ACSL6?	ctg67516 ACSL3?<br>ctg66619ACSL4?
Lamprey	ENSPMAG00000008135 (f) ACSL1<br>ENSPMAG00000004625 (p) ACSL?<br>ENSPMAG00000005099 (p) ACSL?	ENSPMAG00000005133 (p) ACSL3
Coelacanth	ENSLACG00000008655 (f) ACSL1<br>ENSLACG00000012732 (f) ACSL2<br>ENSLACG00000010845 (f) ACSL5<br>ENSLACG00000005834 (f) ACSL6	ENSLACG00000003984 (f) ACSL3<br>ENSLACG00000002977 (f) ACSL4
Spotted Gar	ENSDARP00000053749_1 (f) ACSL2<br>ENSDARP00000123999_1 (f) ACSL5<br>ENSDARP00000039916_1 (f) ACSL6	ENSDARP00000042883_1 (f) ACSL3<br>ENSDARP00000010373_1 (f) ACSL4

Notes: (f) Indicates full sequences (p) Indicates partial sequences

Full sequences were further used in phylogenetic analysis in figure 1 and 2.

Elephant shark

>Cmictg60368ACSL1

METHELINHLRVPELWELRQYIRSLPTYTLMGIGAIAALVTYWYATRTKPPKPACDLSMQSIEVASGERA
RRSALLNSDDLLTRYYEDITTLYEVLKRGLRVSRNGPCLGSRKPNQPYEWMSYTEVSDKAEFLGSGLINQ
GCKATNDQFIGIFAQNRPEWVIVEQACYAYSMVVVPLYDTLGSEAISFILKQAEIAFVFCDTSIKAHSLL
AGVERGQTPLLKVIIVMDPIGNDLQERGRNCGVKIVHFKEVEDEGREQKRPVVPPKPSDLAVVCFTSGTT
GNPKGAMLTHGNIVSNLSAFLKVTEKVGVPTPADSLISFLPLGHMFERIVEAVVLCHGARIGFFQGDIRL
LMDDLKALKPTVLPVVPRLLNRMFDKVHNQANGLIKRQVLEFAAWRKEAQLQNGIVCRDTIWDKLVFHKV
QDNLGGRVKLMVTGAAPVSETVLTFLRAAIGCQFYEGYGQTECTAGCTMSIPGDWTAGHVGAPMPCNIIK
LIDVEDMDYFAVKGEGEVCVKGPNVFKGYLKDPEKTAEALDADGWLRTGDIGKWLPNGTLKIIDRKKHIF
KLAQGEYIAPEKIENVYVRSTPVAQVYVYGESLQACLVGIVVPDPETFSEWAQKTGVRGAYEELCKNQKV
KQAVLEDMVALGKESGLKSFEQVKDIVLHPEMLTVQSGLLTPTFKAKRAELRKYFRSQLDELYANIKM

>Cmictg17226ACSL3

LGQQSKSNIAIFCETRAEWMITAQACFMYNFPLVTLYATLGGNAIAHGLNETQITHIITSKKLLQTKLKD
ILLNVPKLQHIIIVDDKPTAWADYPRGIMVHNMTAVEDLGSKPENLHKQYTKPTTEDIAVIMYTSGSTGI
PKGVMISHGNLIAGIAGMSARVSGLGPKDTYVGYLPLAHVLELSAETLCLACGCRIGYSSPQTLADQSAK
IKKGSKGDTTVLRPTLLAAVPEIMDRIYKNIVNKVEEMNSIKRTLFVLAYNYKMDEIFRGRSTPLCDWLI
FRNVRSLLGGRTRLILCGGAPLSPSTQIFMNICFCCPVGQGYGLTETCGAGTISDVNDYSTGRVGAPLPC
SEIQLKNWEEGGYTVYDRPNPRGEIIIGGPNVSKGYFKNVTKTHEDYFVDKAGQWWFSTGDIGEFYPDGC
LKVIDRKKDLVKLQYGEYVALGKIESALKNSPIIENICAYANSDESYVICFVVPNQKHLMALAKEKNISG
TWSEICKNSDMEREVLNEIIEVAGQAKLETFEIPLKVHLSPEPWTPEMGLVTDAFKLKRKELKNYYEADI
ERMYGGK

>Cmictg24956 ACSl4

CCEIKLRDWEEGGYTCKDHPNPRGEILVGGPNVAMGYFKMNKSNDDFFLDSVGQRWFCTGDVGEFHPDGC
LQIIDRKKDLVKLQAGEYVSLGKVESALKSCSLIDNICAYANSEQSYVISFVVPNQKKLTILAEQKGIEG
VWEELCNNKVMEAEVLREIKEAAQSSKLEKFEVPMKVRLSPE

>Cmictg48116 ACSL6

GEYVAPEKIENIYIRSEPVAQVYVHGDSLQSFLVGIVVPDPEAADAWARKRGFEGPFVELCKAKGLKKAI
M

>Cmictg26023 ACSL4

CVAYGCRIGYSSPQTLSDQSTKIKKGSKGDCSVLKPTLMAAVPEIMDRIYKNVMSKVQEMSYVQRTLFKL
GYDYKLDQIKRGYDAPLCNLLLFKKVKALLGGNVRMMLSGGAPLSPQTQRFMNVCFCCPVGQGYGLTETC
GAGSITEVLDYSTGRVGAPLICCEIKL

>Cmictg18935
ACSL4ACFKYNFPLVTLYATLGEDSVAYGLNESEVTHLITSVDLIDTKLKNVLPKINKLKYLIYVDKKTI
STSGYPEKLRIHSMTCVEELGSKPENLNAHLNSPTPTDLAVVMYTSGSTGHPKGVMINHSNLIAGMTGQC
QRIPWLGPRDTYIGYLPLAHVLELTAEISCVAYGCR

>Cmictg34934 ACSL4

HIIYVDKKTINTSGYPDTLQIHNMAAVEEMGAKSETSEKASSRPAPSDLAVVMYTSGSTGRPKGVMMIHS
NLIAGMTGQCQRIPELGPKDTYIGYLPLAHVLELTAEISCVAYGCR>Cmictg79717 ACSL3

EIGLGIPGEPLFRSQGWFCTGDVGEFHHDGCLKVIDRKKDLVKLQAGEYVALGKVESALKNSPLIDNICV
YANSDQSYVICFVVPNQKQLLALAHQ

Little skate

>Lskctg17167ACSL1?

LANRLYDKIHSQANGFFKRKILQLASWRKIAELHKGIMRRDSIWDFLVFRKIQDSLGGQVKLMVIGAAPV
SDTVLDFVRAAMGCQFYEGFGQTECTAGCSMTIPGDWSAGHVGAPMPCNLVKLIDVHEMNYFSANGEGEV
CVKGPNVFSGYLKDPERTGEVLDETRWLYTGDIGKWLPNGTLKIIDRKKHIFKLAQGEYIAPEKIENIYV
RCELIAQVFVHGDGLQAFLVGIVVPDPEVVPEWANKWNLKGSYEELCKSQALKDAILANMLKVGKEAGLK
TFEQVKDIHLYPEMLTVESGLLTPTFKTKRLEMRKFFHKQIAELYAQNAV

>Lskctg11347ACSl1?

MQTVDLMTHLRMPELGEFQQYLRAVPTYSILGIGTIAALTMYWYTSKPKAQIPPCDLSMQSIEVDNFDHS
RKSVLLKEGQDLMTVYYQEVQTLYDVLKRGLIVSGNGPCVGCRKPNQPFQWISYREVSERAECIGSGFIR
RGYKGGNSEYIGIYAQNRPEWVIVEQACYAFSMVVVPLYDTLGIEAISFIINKADMEVVVCDTAGRARML
LNGVETGITAELKTIVVMESFGEDLVHRGIRHEVEVVSLAEIEKSGKEKKHRTSPPDPKDLAVICFTSGT
TGKPKGAMLTHKNIVANFSAFVKVTEGQWTASPSDIHISFLPLAHMFERLVQTVVLCHGGRIGFFQGDIR
LLMDDIKLLKPTIFPIVPR

>Lskctg31550ACSL5?

VEDMNYFAAKGEGEVCVKGTNVFKGYLKDPEKTLEAIDSDGWLHTGDVGKWLPNGTLKIIDRKKNIFKLA
QGEYIAPEKIENIYIQSGLVTQVFVAGDSLQS

>Lskctg34321ACSl5?

NSAACFRMMGSSISINVDDVSISYLPLAHMFERVVQTALYCSGSRVGFFQGNIQLLLDDMKTLQPTFFPV
VPRLLTRVYDKVQSS

>Lskctg43339ACSL5?

LKDDKVISHFYDDVKTLYEAFQRGLRVSGNGPCLGYRKPNQPYQWLSYKQVIDRAEHLGSGLLHRGCKPS
P

>Lskctg82046ACSL2?

ELAIVICDNMNKVKVLLGNCELKQTPSLSTIILMDPFDDALKERGTKCHVEVLTLKEVEDLGRENLQKPI
PPKPDDLSIVCFTSG

>LSKctg12475ACSL3?

MHFGSGLAVLGQRPKFNIAIFCETRAEWMIAAQSCFMYNFPLVTLYATLGGQAIAHGLNETEVTHIITSK
KLLQTKLKEILLHVPKLQHIIIVDDKTTAWADYPRGIMVHNMTGVEALGAKPENLNRARMRPTTLDIAVI
MYTSGSTGLPKGVMISHSNLVAGIAGMVARVPDLGPKDTYIGYLPLAHVLELSAETLCMACGCCIGYSSP
QTLADQSAKIKKGSKGDTTVLKPTLLAAVPEIMDRIYKNIVTKVEDMNSIQRTLFVVAYNYKQEQIAVGY
NTPICDWLIFRKIRLLLGGKTRLILCGGAPLSPSTQTFMNICFCCPVGQGYGLTETCGAGTISDVCDFTT
GRVGAPLPCCEITLKNWEEGGYTVYDKPHPRGEIVIGGPNVTLGYFKNKSKTMEDYFLDKNGQAWFCTGD
VGEFHHDGCLKVIDRKKDLVKLQAGEYVALGKVESALKNSPLIDNICVYANSDQSYVICF

>LSKctg14318ACSL4

MAKRIKAKATSDKPGSPFRSVDHFDSLASVDFPGLDTLDKLFDAAVKKFPEHDCLGTRERLSEENEVQAN
GKVFKKLILGEYRWLSYEDVGQRVNSFGRGLSALGLKPKDKIAIFCETRAEWMIAAQACFKFNFPLVTIY
ATLGEDAVTYGLNECEVTHLVTSMELLDTKLKSVLPKIRHLKHIIYVDKKTINTSGYPDTLQIHNMAAVE

EMGAKSETSEKASSRPAPSDLAVVMYTSGSTGRPKGVMMIHSNLIAGMTGQCQRIPELGPKDTYIGYLPL
AHVLELTAEISCVAYGCRIGYSSPQTLSDQSTKIKKGSKGDCTVLKPTLMAAVPEIMDRIYKNLMSKVQE
MNYLQRTLFKLGYDYKLEQIQRGYDAPLCNLVLFKKVKALLGGNVRMMLSGGAPLSSQTQRFMNVCFCCP
VGQGYGLTETCGAGSITEVSDYSTGRVGAPLICCEIKLRDWEEGGYTTHDQPNPRGEILIGGPNVAEGYF
KGNSTNDDFFEDSSGQRWFCTGDVGEFHSDGCLQIIDRKKDLVKLQAGEYVSLGKVESALKSCSLIDNIC
AYANSEQSYVISFVVPNQKKLTALAEQKRVQGTWEDICNDSKMEAEVLREIKEASASSKLEKFEVPIKVR
LSPEPWTPETGLVTDAFKLKRKELKNHYLNDIERMYGGK

Coelacanth

>LmaACSl1ENSLACG00000008655

MTTMMQAHDLLRHLRLPELGEVREYVRTLPTNTLMGFGAFAALTTYWYATRPKALKPPCDLSLQSVEIEG
SNGARRSALLETDQPLSFVYEDAQTMYELFQRGLRVSGNGPCLGFRKPNQPYEWISYQEVADGAEFLGSG
LIHRGCRPAPDQFIGIFAQNRPEWIITELACYTYSMVAVPLYDTLGAEAISYIINKAEIATVVCDEPDKA
RVLLENVEGGETPFLKMIVLMGHFENDLVERGKKCGVDILSMKAVEDLGKVNLCKPVLPKPSDLAVVCFT
SGTTGHPKGAMLTHGNIISNFSAFVKVTEKAVFPNTDDILISFLPLAHMFERIVEAVILCHGARIGFFQG
DIRLLMDDLKTLQPTIFPVVPRLLNRMFDRIFAQASTPIKRWLLEFAAKRKEAELRSGIVRSDSVWDKLI
FNKVQASLGGRVRLMVTGAAPISPSVLTFLRAALGCQFYEGYGQTEGTAGCTLTIPGDWTAGHVGAPMPC
NIIKLVDVEEMNYFSAKGEGEVCIKGTNVFQGYLKDPERTAEAFDKDGWLHTGDIGKWLPNGTLKIVDRK
KHIFKLAQGEYIAPEKIENTYVRSEPVAQVFVHGESLQAYLVGIVVPDPEVLPSWASKRGIEGSFAELCK
SRELKNTIIEDLVKLGKEAGLKSFEQVKDIMLYPEMFSIQNGLLTPTLKAKRPELRKYFKSQLDELYENI
KM

>LmaACSL2ENSLACG00000012732

IMESIEGLLDALHVPEYLGIPELEDASNFILSFSTPTLILIGAAVFALVYWLAMRPKATKLLCDLNNQSL
PVQGEPACRRSTLMKEDQYYCDDVKTAYEAFQRGVRISGDGPCLGFRKLGSPYNWISYKEVSSRAEFFGS
GLLHRGCSPSPDQFIAVFSQNRPEWVISKLACYTYSMVIAPLYETLGTEGLIHILNTTESSTVICDKPGR
AETLLSYVEGSKTPYVKTIILMEPFEDSLGGRGRSCGVDVLSMKDVEDLGKENWKAPMPPKPEDIAVICS
TSGTTGKPKLAMLSHKGMVCCISSVLSILQKKITPRAEDTILSFLPLAHVYELMAQLMFYCQGGRVGFYQ
GDIQLLMDDCRTLKPTFFPTVPRILNRMYDQIHSAMKSPVKRFLLHCAVWAKQVELRRGIIQNNSIWDWL
LFSRIQAILGGRVRIILTGSAPISSTVLSFFRTTLGCLIVEGYGQTECTGACTCSLPGDYTAGGHVGPPV
SWSTVKLEDVEEMNYFVSNGEGEICVKGPGVFLGYLKDTEKTAEAIDADGWLHTGDVGRWLPNGTLQIID
RKKHLFKLSQGEYIAPEKIENAYLRCTTVSQVFVHGESLQSFLVGIVVPDPEVLPSFAEKRGITGTYEEL
CRNPEVRKAVLEDMTRIGREAGLNSLEQVKTIYLHPEMFTIAKGMLTPTLKSIRAKLRNYFQQQINQLYT
NPSL

>LmaACSL6ENSLACG00000005834

FFFFFFLLHGTLFLMTEFAASALEKMQAQEILRSLRLPEFDEFSQFFRSMSAPTLVGIGAFAVVVAYWLA
SRPKAVRPPCDLAEQSQEVPGCDGAHRSVLGDTPQLLTHYYDDARTMYEVFQRGLHISENGPCLGFRRPK
QPYQWLSYKEVSNRAECLGSGLLQQGCKPSTDQFIGVFAQNRPEWIISELACYTYSMVVVPLYDTLGPGA
IRYIINTAEISTVICDKLEKARVLLEHVEKQETPGLKTVILMDPFHEDLVERGRKCGVHIQSMKEVEDLG
RANRRVPMPPRPEDLSIVCFTSGTTGNPKGAMLTHGNVVADFSGFLKVTEKVIFPRQDDVLISFLPLAHM
FERVIQSLWSPTCEDVHISYLPLAHMFERMVQSVVYCHGGRIGFFQGDIRLLSDDMKALRPTIFPVVPRL
LNRMYDKIFSQADTPFKRWLLEFAAKRKEAEVRNCIIRNDSLWDKLFFNKIQASLGGRVRMIVTGAAPAS
PTVLGFLRAALGCQVYEGYGQTECTAGCTFTTPGDWTSGHVGAPLPCNLIKLTDVQEMNYFATKGEGEIC
VKGPNVFKGYLKDLEKTAEALDENGWLHTGDIGKWLSNGTLKIIDRKKHIFKLAQGEYIAPEKIENIYIR
SEPVAQLYVHGDSLQSCLVGIVVPDPEVMPTWAKKRGFEGSYAEMCKNKELKIAIMQDMVRLGKESGLHS
FEQVKDIYIHSEMFSVQNGLLTPTLKAKRTELREYFKKQIEELYANVSM

>LmaACSL5ENSLACG00000010845

MNFLLHFLFSPLSTPALIGIFSFGAAIFIWLITRPKPVRPPVDLNNQSVGLKEGARRSALLKSDKLISYY
YDDARTLYEIFQRGLHISGAGPCLGYRKPKQPYQWLTYKQVSDRAEYLGSGLLHRGCRPAPDQFIGIFAQ
NRPEWIISEFACYTYSMVAVPLYDTLGPEALIFIINRAEISTVICDKPSKAITLLDNCEERQTPGLNTII
LMDPFEDDLKEKGAKFGVEILTLQQVEDLGKENLRKPIPPKPEDLSIVCFTSGTTGDPKGAMLTHENVVA
DSAAFIKSLESTFAPLQEDISISYLPLAHMFERVVQTIMYSSGAKVGFFQGDIRQLPDDMMALQPTVFPV
VPRLLNRIYDKVQSGAQTPFKKWLLNFAVARKHAEVKQGIIRNDSIWDNLIFHKVQATIGGRARVMVTGA
APISPTVLKFLRAVLGCQIFEAYGQTECTAGCTFSIAGDWTTGHVGAPLPCNLVKLVDVEEMNYFAVNGE
GEVCIKGTNVFKGYLKDSEKTAEALDADGWLHTGDVGKWMSDGTLKIIDRKKNIFKLAQGEYIAPEKIEN
VYIRSSPVAQVFVHGDSLQSCLVGIVVPDPEVLPDFAAKLGQNGSYEELCKNPVVRKAILEDMVKLGQKA
GLKSFEQVKEIYIYTEMFTIENGLLTPTLKAKRAELSKYFKGQIDSLYASMQG

>LmaACSL3ENSLACG00000003984

MKLEEDLHPVCLYIIHFLIKVYTCITFLPWYYFSGASQNLAKAQQVKARPVQNCPGKPYRSVNSLHCLAS
VLYPGCDTLDKVFEYAKNKFKDEDCLGTREVLNEDDEIQPNGRVFKKVILGRYNWLSYEDAYVKAALFGN
GLAQLGQMPRCTIAIFCETRAEWMIAAQACFMYNFPLVTLYATLGSRAIAHGLKESAVSHIITSKELLQT
KLKPIVTDVPQLKHIIIVDEKPTAWTGYPSGITVHSMAAVQALGTKPEDLNVPHRRPKPSDIAVIMYTSG
STGLPKGVVISHSNLIAGITGMAERIPNLGVKDTYVGYLPLAHVLELSAELVCLSHGCRIGYSSPQTLAD
QSTKIKKGSKGDTSVLKPTLMAAVPEIMDRIYKNVMNKVNEMNRFQRNLFILAYNYKMDQISKGCSTPLC
DSLVFRKVRSLLGGKTRVILSGGAPLSPATQRFINICFCCPVGQGYGLTETCGAGTISEVWDYSTGRVGA
PLVCCEIKLKDWEEGGYYNTDKPHPRGEILIGGQNVTMGYFMNAEKTKEDFLVDLDGQRWFCTGDIGEFH
PDGCLKIIDRKKDLVKLQAGEYVSLGKVEAALKNCPLIDNICAYANSDQSYVIGFVVPNQKELLYLSAQK
GIKGTWEEICNNHEMEKEVLKVIADVAISAKLEKFEVPVKVRLSAEPWTPETGLVTDAFKLKRKELKSHY
QEDIERMYGGK

>LmaACSL4ENSLACG00000002977

TMKLKVLKVSSILLLPVYVLMFVYTILTFIPWYFLTNAKKKKAMAKRIKAKPISEEPGSPYRSVDHFNSL
ATIGIPGADTLDKLLDQAVVKFGKKDCLGTREFLSEENEVQPNGKVFKKLILGEYKWISYEEVHKQVTHF
GSGLAALGQKPKNTIAIFCETRAEWMISAQACFKHNFPVVTLYATLGEQAVAYGLKESQVTHLITSIELL
ETKLKRVLSNIPNLKHVIYVDKKNFDKSGYPAGIQIHSMASVTELGAKPENLNSPITHPVLSDLAVVMYT
SGSTGLPKGVMMVHSNLIAGMAGQCNKIPELGPKDTYIGYLPLAHVLEMTAEISCIAYGCRIGYSSPQTL
SDQSTKIKKGSKGDCTVLKPTLMAAVPEIMDRIYKNVMSKVQEMSYVQKTLFKLGYDYKLEQIKRGYDAP
LCNLFLFKKVKALLGGNVRMMLSGGAPLSPQTQRFMNICFCCPVGQGYGLTETCGAGTITEATDYSTGRV
GAPLICCEIKLRDWPEGGYTTRDKPNPRGEIVIGGPNVSMGYFKSEEKTSEDFIIDRNGQRWFCSGDIGE
FHPDGCLQIIDRKKDLVKLQAGEYVSLGKVEAALKNCALIDNICVYANSYQSYVISFVVPNQKKLMALAQ
HKGIEGSWEELCNNPTMEAEVLKAITDVASSVKLERFEIPVKVRLSPDPWTPETGLVTDAFKLKRKELKN
HYLNDIERMYGGK

Spotted Gar

>Loc_ACSL2_ENSDARP00000053749_1

SLSPSSLLGLGALASLTAYWLATRPRPIRPPCDLHAQSVPVQGDPSCRRSALLQDEMLLEFYYEDTRTAY
EMFQRGLRVSGDGPCLGFRKPSEPYKWISYREVCEQAQALGSGLLARGCQPNPQQFIGIFAQNRPEWVIA
ELACYSFSMAVVPLYDTLGQEAMVHILNIAEITMVICDKPEKAESLLSHKEQTLAPLLGSIVLMTPCSAA
LLERAKKCGIEILQFSELMVSQRECVLVILPPKPEDLAVVCFTSGTTGKPKGAMITHGNIASNTSSVIKI
LEGSFVIRQEDISISYLPLAHMFERMIQVSMFCHGARVGFYQGDLSLLMDDIKTLRPTFFPVVPRLLNRI

YDKILASVSSPLKRALLHYAVRRKQAELSSGVVRNNSVWDRLIFNKIQASLGGNLRFILTASAPISPTVL
SFLRATLGCLIFEGYGQTECTAGCTFSMPGDWTAGHVGAPLPCAMVKVTDIPEMSYYSHNGEGEICIKGH
SVFRGYLRDHERTAEALDPEGWLHTGDVGKWLPNGALQIIDRKKHIFKLSQGEYIAPEKIENVYIRSAPV
LQVFVHGDSLQSHLVGIVVPDPEVFTDWAKERGIVGSYEELCRNPDVKKAVLEDMTVIGKEAGLKSFEQV

>Loc_ACSL6_ENSDARP00000039916_1?

PPQPDDLSIVCFTSGTTGNPKGVMLTHGNVVADFSGFLKVTEKVIFPRQDDVLISFLPLAHMFERLIQSV
VYCHGGRIGFFQGDIRLLSDDMKALCPTIFPVVPRLLNRMYDKIFSQASNPVKRWLLEFAARRKSAEVHS
GIIRNDSVWDKIFFSKIQASLGGRVRMIVTGAAPASPTVLGFLRAALGCQVYEGYGQTECTAGCTFTTPG
DWTSGHVGAPLPCNLIKLVDVADMNYFASKGEGEVCVKGPNVFKGYLKDQEKTAEALDEDGWLHTGDIGK
WLPNGTLKIIDRKKHIFKLAQGEYIAPEKIENIYIRSEPVAQLYVHGDSLQSCLVGIVVLDPEVLLDWAR
KRGIEECFGDLCKNKEVKKAVMDDMVRLGKASGLHSFEQVKDIYIHNELFSIQNGLLTPTLKAKRSELRE
FFKEKIEHMYANISM

>Loc_ACSL5_ENSDARP00000123999_1

MSCILQFLFSPLPTPAIAGLFTFGAGILIWLVTRPKPIKLPVDLNRQTVGIKDGARRSALLKDDKLMSYY
YEDARTLYEVFQRGLHVSGNGPCLGYRKPRQPYQWLKYKQVSDRAEYLGSGLLHRGLKPSPDQFIGIFAQ
NRPEWIISELACYTYSMVAVPLYDTLGPEALVYIVNKAEISTVICDKPDKAAILLTNCEKGLTPVLNTIV
LMDPFSTDLKDRGMNCGVEILALKEVEV*GSKFCFATEIPPKPEDLSIVCFTSGTTGDPKGAMLTHENVV
ADAAGFIKSTESAFGPVPQDVSISFLPLAHMFERVVQTVMYSSGAKVGFFQGDIKLLPDDMKALRPTVFP
VVPRLLNRVYDKVQSGAQTPFKKWLLNFAIERKHSEVKQGIIRNDSIWDKLIFHKVQETMGGRVRVMVTG
AAPISPSVLSFLRACLGCQIFEAYGQTECTAGCTFSMPGDWTTGHVGVPVPCNIVKLVDVEEMNYFAANG
EGEVCIKGRNVFKGYLKDPEKTSEAIDENGWLHTGDIGKWLPSGVLKIIDRKKNIFKLAQGEYIAPEKIE
NVYVRSEPVAQVFVHGDSLQSCLVGIVVPDIEVLPDFAVKLGIKGSYKELCINKEIKKAILADMVRLGKQ
AGLKSFEQVKDLYLYPEQFTIENGLLTPTLKAKRAELTKFFKDQIDSLYA

>LocACSL4_ENSDARP00000010373_1

EVHSILLLPVHLFMWLYTLLTFIPWYFLTDTRKKKTMAKRIKAKSTTGKAEGPYRSVDHFQSLATMDFEG
KDTLDKLFDHAVQRFGKADCLGTREVLTEENETQPTGKVFKKLILGEYKWLTYEDVNRQVTLFGSGIAAL
GQQPKNTIAIFCETRAEWMIAAQACFRRNFPVVTLYATLGEDAVAYGLNESGVTHLITSVELLETKLKKV
LSEIKNLKHIICVDKKNASKTGYPEGLHIHSMESVQELGGKPENLSVTPCHPQPTDLAVVMYTSGSTGRP
KGVMIIHSNLIAGMTGQCERIPGLGPKDTYIGYLPLAHVLEMTAEISCVTYGCRIGYSSPQTLSDQSTKI
KKGSKGDCSVLKPTLMAAVPEIMDRIYKNVMSKVQEMSYVQRTFFKLGYNYKLEQIKMGYDAPLCNLLFK
KVKALLGGSVRMMLCGGAPLSSATQRFMNICFCCPVGQGYGLTETCGAGTITEVADYSTGRVGAPLICCE
IKLRDWAEGGYTNQDVPHPRGEILIGGPNVTMGYYKNGQINEDFFVDENGQRWFCTGDIGEIHADGCLQI
VDRKKDLVKLQAGEYVSLGKVESALKNCSLIDNICAYANSDQNYVISFVVPNQKRLTALANKQGISGAWE
DICNHPAMESEVLKEIKEVATSIKLQRFEIPVKVRLSPEPWTPETGLVTDAFKLKRKELKNHYLNDIERM
YGGK

>LocACSL3ENSDARP00000042883_1

LLSGASQNLERAKRVKARPVNNQPGGPYRSVNSMSCLASSLYPGCDTLDKVFEYAKNKFSTNHCLGTREL
LSEEDEVQPNGKVFKKVILGEYRWLSYKETHLAAARFGSGLAALGQKPKSTIAIFCETRAEWLITAQACF
MYNFPLVTLYATLGGPAIVHGLNETEVTHIITSKDLLQSRLKAILLEVPRLQHIIIVDDKSSTWIDYPRG
ITIHNMAEVQALGSKEENMSKPRCQPAPSDIAVIMYTSGSTGIPKGVMISHSNLIAGITGMAERIPDLGE
NDTYIGYLPLAHVLELSAELVCVSHGCRIGYSSPQTLADQSTKIKKGSKGDTSVLKPTLMAAVPEIMDRI
YKNVMRKVEEMNSVQRTLFVLAYNYKMEQISKGYSTPLCDSFVFRKVRSLLGGRTRVLLSGGAPLSAATQ
RFMNICFCCPVGQGYGLTETCGAGTISEMWDYSTGRVGAPLVCSEIKLKSWEEGGYYCTDKPNPRGEILV
GGPNVTMGYYKNEAKNKDDFFVDEKGQRWFCTGDIGEFHPDGCLKIIDRKKDLVKLQAGEYVSLGKVEAV
LKNCPLIDNICAYANSDQSYVIGFVVPNQKQLMALAEHKKVGGTWEEICNNSEMEKEVLRVMAEAASGAK
LEKFEIPMKIRLSAEPWTPETGLVTDAFKLKRKELKTHYQDDIERMYGGK

Small spotted catshark

>SSCctg18625ACSL1?

VVFCDTAVKAEAVLLGVEKGQTPDIKTIIIMDPFGAELKNRGKAYGVEIVSLNIIETAGREMKRDPRLPQ
PSDLAVICFTSGTTGNPKGAMLTHRNIVSNFSAFVKVTEGQWVASPSDIHISFLPLAHMFERLVQIVVLC
HGARIGFFQGDIRLLMDDIKVLQPTIFPVVPRLLNRMYDKVHSQANSFLKRQILALATWRKTAELRKGIM
RRNSIWDKLVFHKIQESLGGKVRLMVTGAAPVSDTVLTFIRAAVGCQFYEGYGQTECTAGCSMTIPGDWS
AGHVGAPMPCNLIKLVDIEEMNYFAREGEGEICIKGSNVFLGYLKDPEKSAEALDQRGWLHTGRGQHGNG

>SSCctg67516ACSL3

MKLKKGVNPIFSLFLQCVIVVCNLLFILPLQLFAGSRRRPSIRAKSISNHPAGPYRCVESLDRLLASLYP
GADTLDKIFQFATDGFRHKNCLGTREILSEEDEIQPSGRVFKKLILGHYKWLTYDEVYRRVAHFGSGLAM
LGQRPKANIAIFCETRAEWMIAAQSCFMYNFPLVTLYATLGGQAIAHGLNETEVTHIITSKKLLQTKLKE
ILLNVPKLQHIIIVDDKPTAWSEYPRGIMVHNMAAVEALGSKPENLNRVRAKPTSLDIAVIMYTSGSTGL
PKGVMISHSNLIAGIAGMCSRVPDLGPKDTYIGYLPLAHVLELSAETLCMACGCGIGYSSPQTLADQSAK
IKKGSKGDTTVLKPTLLAAVPEIMDRIYKNIVTKIEDMSSMQRTLFVVAYNYKMEQMSLGCSTPICDWLI
FGKIRSLLGGKTRLILCGGAPLSPSTQTFMNICFCCPVGQGYGLTETCGAGTISDVFDYTTGRVGAPLPC
SEITLKNWEEGGYTVYDKPHARGEILIGGPNVTLGYFKNKSKTLEDYFVDKDGQAWFCTGDIGEFQDDGC
LKVIDRKKDLVKLQAGEYVALGKVESALKNSPLIDNICAYANSDQSYVICFVVPNQKQLLALAQQKGIIG
SWNDICNRPEMEKEVLREITDAAAVARLEKFEIPLKVRLSPEPWTPETGLVTDAFKLKRKELKTHYLADI
ERMYGGK

>SSCctg66619ACSL4

MAKRLKAKATSEKPGSPFRSVDHFDSLAKMDFPGLDTVDKLFEAAEKKFRKQHCLGTRELLSEENEVQVN
GKVFKKLILGEYKWLSYEEVNQHVNCFGSGLTALGLKAKDMIGIFCETRAEWMIAAQACFKYNFPLVTIY
STLGEDAVAYGLNESEITHLITSAELLDTKLKKVLPKIQMLKHIIYVDNKVINTSGYSETLQIHSMESVE
ELGAKPENMDITPSRPVPSDLAVVMYTSGSTGHPKGVMMIHSNLIAGMTGQCQRIPGLGPKDTYIGYLPL
AHVLELTAEISCVAYGCRIGYSSPQTLSDQSTKIKKGSKGDCSILRPTLMAAVPEIMDRIYKNVMSKVQE
MNYVQRTLFKLGYDYKLEQIKRGYDAPLCNMLLFKKVKSLLGGNVRMMLSGGAPLSPQTQRFMNVCFCCP
VGQGYGLTETCGAGSITEVLDYSTGRVGAPLICCEIKLRDWEEGGYTTNDQPHPRGEILIGGPNVAMGYF
KLNKSSHDFFEDNTGQRWFCTGDVGEFHPDGCLQIIDRKKDLVKLQAGEYVSLGKVESALKSCSLIDNIC
AYANSEQSYVISFVVPNQKKLTALAEQKQVQGTWEEICNDSKMEAEVLREIKEASASSKLEKFEVPVKVR
LSPEPWTPETGLVTDAFKLKRKELKNHYLNDIERMYGGK

>SSCctg94905ACSL1?

MQASELLTQLRIPEFGEVRRFICSLPPSTLIGIGTIAALVAYWYATRAKAQKPPCDLSKQSVEVEGGERA
RRSVLLKSDEPMVFYYLDVKTLYDVLKRGLWVSDNGPCLGFRNPDQPYQWLSYREVITRAEFVGSGLFTR
GYKPGNDQFIGIFAQNRPEWVIIEQACYTYSMVVVPLYDTLGDEAISYILNKADIAVVFCDTA

>SSCCtg81762ACSL6?

MDPFEMDLVEMGNDCGVQILALQEVENLGRVNRQTPVPPRPEDLSIVCFTSGTKGKPK

>SSCctg63419 possible Acsl2

CFRSLDCSKKNCRSSPDQFIGVFAQNRPEWIIAELACYTYSMVIVPLYDTLGPEAIRYIINTAEISTVIC

Lamprey

>PmaASCL3ENSPMAG00000005133?

QAILMQVPRLRYVVLVDGTASGVAGVKLPRGIEVMGMSDVEELGDKPQHRQRARDRPGPRDLAVVMYTSG
STGIPKGVRIAHSNLVAAITGMYLRINDICGDDTYIGYLPLAHVLELGAEMVCLSRGCRIGYSSAQTLTD
QSTRIKKGSQGDVTILRPTLMAAVPEIMERIYKGVMGKVQCMSLLQRIIFKLAYNYKLEQLERGFDTPLC
NRLVFNKLCALLGGNVRLLLSGAAPLSPRTQRFMNVCFCCPVGQGYGLTETCAAGTIAELQDYSTGHVGA
PLSCCEIQLRNWEEG

>PmaACSL1ENSPMAG00000008135

MQSAQEVLKTLRVPELDEVRQYVRSLPAPALMGLGALGTMTAYWLATRPRALSAPCDLSKQSLPVKGREY
QRRSPLVPDDDTFFTYFYEDARTGYEVFQRGLRISNNGPCLGYRKPNHPYEWISYKETSDRAEYLGSGFL
HLGAKPSSEQVIGIFSQNRPEWIIAEQACYTYSLVVVPLYDTLGRESIDYIINQAEISMVVCDKLEKVKG
LLESIEEGAIRIVKTIVVMDPFDGVMEQRARKCGIDLILFRELEVIGKTNHREPIPPQPDDIAIICYTSG
TTGNPKGAMLTHKNMISDFSAFLAITKDTFLPNTDDVLISFLPLAHMFERLVEASILCNGGRVGFYQGDI
RLLMDDMKVLQPTVFPVVPRLLNRIHDKVLSGAKSHFKRWLLEFAVSRKIAELRCGVVRKDSIWDKLIFH
RVQASMGGRVRFMVTGAAPISASILTFLRAILGCQVYEGFGQTECTAGCTFTMPSDSTAGHVGPPMPCNH
IKVVDVAEMNYFAANGEGEVCVYGTNVFKGYLKDPTRTAEALDEDGWLHTGDIGKWLPNGTLKIVDRKKH
IFKLSQGEYIAPEKIETVYVRSEPVAQVFVHGDSLQSCLVCIVVPDMEVLPSWVQKRGIKCNPNSVFINK
DVRAAILHDMVRLGKEAGLKSFEQVRAVHLHSDLFSIENGLLTPTFKVKRAEVCKFFHSEIDSLYAGITV

>PmaACSL1ENSPMAG00000004625?

SLQDCGRMHKRTTLPPKPEDLAIVCFTSGTTGNPKGAMLTHRNIVSDMSGFLKVTESLFLPETSDIAISY
LPLAHMFERLVQATLFCHGASIGFFQGDVRLLLDDMQALRPTVFPVVPRILNRMYDKVLGSTRTPFRRKL
LEFGARRKMAELQRGVVRRNSLWDWLVFRPMQLSVGGRVRMIMTGAAPISPGVLNFLRVVMGCQMYEGYG
QTECTAGCTLTLPGDWKAGHVGAPMPCNYIKLHDVKDMEYYTSQGKGEVCVKGPNVFKGYLKDAEKTAEA
VDRDGWLHTGDIGQWMPNGTLKIIDRKKHIFKLAQGEYIAPEKIENVYSRCEPVAQVYVHGDSLQACLVA
VVVPDPEILCCWIRKKGIVGTYSELCRNKEVRQAILEDMQRLGKESDLQPFEQVKDVHLHNEMFSIENGL
LTPTFKAKRTELRTHFRSVISSMYQNVKA

>PmaENSPMAG00000005099ACSL1?

VEEYIPWRTQMLSIGGLLENVWGSMPPSAWLGVGTASLFTGYWYLSRPRPICPPCQLTQQSVEVEGQDGV
RRSALLKKDKLLEFYYEDAKTMYEVFHRGMRVSKNGPCLGYRKPKKPYQWMSYKEVAERSEWFGSGLIHK
GCRPATDQFIGVFAQNRPEWIITEQACYMYSMVVVPLYDTLGQEAIRFIINRAAEIAVVVCDTVERARVL
LRGVENRETPGLRTVVVMETPDLGLIQWGITCRVDVLSFQYIE
