# Supplementary material for: Diversity and history of the long-chain acyl-CoA synthetase (Acsl) gene family in vertebrates
Source: BMC Evol Biol. 2013 Dec 12;13:271. doi: 10.1186/1471-2148-13-271 (PMC3890633; doi:10.1186/1471-2148-13-271)
Supplement: Additional file 2 — Partial Acsl gene annotations in green spotted pufferfish. [file 1471-2148-13-271-S2.pdf]

Additional file 2

Partial *Acsl* gene annotations in green spotted pufferfish

A- Genomic location of the partial sequences of *Acsl*1 from green spotted pufferfish

Acsl1a - ENSTNIG00000018054 (complete sequence)
Acsl1b- ENSTNIG00000000345 (partial sequence)
Acsl1c- ENSTNIG00000010115 (partial sequence)

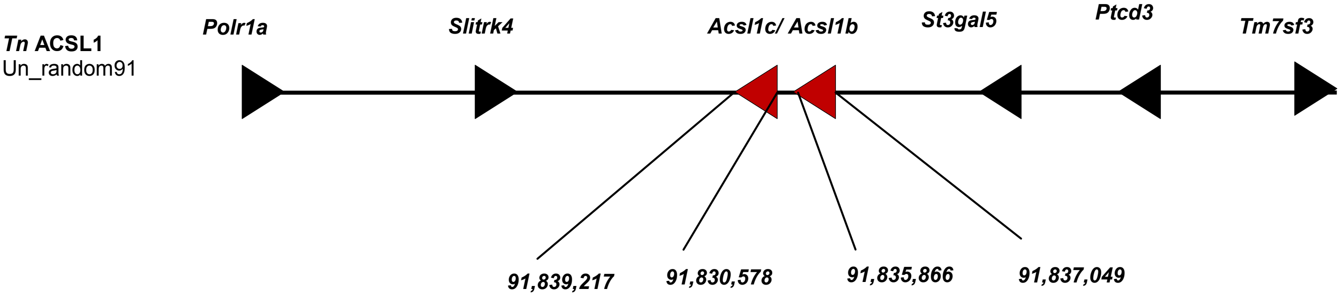

B- Alignment of the partial *Acsl1b* and *Acsl1c* sequences and complete *Acsl1a* sequence

Highlighting in grey marks the overlapping region between the two partial sequences

```
ACSL1a_ENSTNIG00000018054      MQTPEALKQFWIPELDNIQHFLGGMSGNALVGMGVLVALTTYWLASRHRA
ACSL1b_ENSTNIG00000000345      IFIMDLVYRLGLLSLDSVTQYVRSVSTPVWVGTGLVAAATTYLLTARPKA
                               :   : : :: : .**.: ::: .:*  . ** *::.* *** *::* :*

ACSL1a_ENSTNIG00000018054      VKQRVDFSRQSVELPGGEGIRRSVLVENDQLITHYYDDARTFYELFLRGL
ACSL1b_ENSTNIG00000000345      LPPICDLDMQSIEIPGGELARRSALQNGDAYTKCYYDDARTMYESFLRGL
                               :    *:. **:*:****  ***.* :.*   . *******:** *****

ACSL1a_ENSTNIG00000018054      RESNNGPCLGSRKLNHPYEWQSYQEVVADRAKHIGSALLNKGHSHTGDKF
ACSL1b_ENSTNIG00000000345      RVSNDGPCLGSRKPKQPYEWLSYSEVK-ERAENLGSAFLHRGHSKTKDPH
                               * **:******** ::**** **.**  :**:::***:*::***:* * .

ACSL1a_ENSTNIG00000018054      IGIFSLNRPEWTISELACYTYSLVAVPLYDTLGREAIGYIIDKATISTLI
ACSL1b_ENSTNIG00000000345      IGIFSQNRAEWTISELACYTYSLVSVPLYDTLGTEAIIYIVEKASISTIV
                               ***** **.**************:******** *** **::**:***::
```

```
ACSL1a_ENSTNIG00000018054      CDLPEKAWMVLDCINGKGKSVKRIVIMGPFQSELVERAEECDIEIISFED
ACSL1b_ENSTNIG00000000345      CDLSSKVDLLLSCLEDKKHAVKTVVLMEKPSVELVSRAKRSGIDVISVEE
                               ***..*. ::*.*::.* ::** :*:*   . ***.**:...*::**.*:


ACSL1a_ENSTNIG00000018054      FEALGQDTVMEPVPPAPEDLALVCFTSGTTGKPKGAMLTHGNIIANTAAF
ACSL1b_ENSTNIG00000000345      MEALGKANRQPPVPPKPEDMAVICFTSGTTG-------------------
                               :****: .   **** ***:*::********

ACSL1a_ENSTNIG00000018054      LKLTEKDCMLCVHDIHISYLPLAHMLERVIHGVVLVHGGRVGFFQGDIRL
ACSL1b_ENSTNIG00000000345      --------------------------------------------------

ACSL1a_ENSTNIG00000018054      LMDDLQTLKPTVFPMVPRLLNRMCDKIFSQADTPLKKWLLRLAFSRKIAE
ACSL1b_ENSTNIG00000000345      --------------------------------------------------

ACSL1a_ENSTNIG00000018054      LNQGVVRQDTIWDRLIFKKVQANTGGRVRMMITGAPPVCPKNLTYINITT
ACSL1b_ENSTNIG00000000345      --------------------------------------------------

ACSL1a_ENSTNIG00000018054      MLQLYEGYGQTECTAGCSMSLPGDWIAGAVGPPVPCNDIKLVDVAEMNYF
ACSL1b_ENSTNIG00000000345      --------------------------------------------------

ACSL1a_ENSTNIG00000018054      AANGEGEVCAKGTNVFKGYLGDAEKTAEALDEDGWLHTGDIGKWLPNGTL
ACSL1b_ENSTNIG00000000345      --------------------------------------------------

ACSL1a_ENSTNIG00000018054      KITDRKKNIFKMAQGEYIAPERIEMIYNRSEPVAQIFVHGDSLKACLVAI
ACSL1b_ENSTNIG00000000345      --------------------------------------------------

ACSL1a_ENSTNIG00000018054      VVPDSETLPDWIKKKGIEGPPTGLCKNQDVKRAIQEDILRLGREAGLKSF
ACSL1b_ENSTNIG00000000345      --------------------------------------------------

ACSL1a_ENSTNIG00000018054      EQVKDITLHPEMFSIQNGLLTPTLKSKRVELRRYFRKQIDEMYAKIKR
ACSL1b_ENSTNIG00000000345      ------------------------------------------------
```

```
ACSL1c_ENSTNIG00000010115      --------------------------------------------------
ACSL1a_ENSTNIG00000018054      MQTPEALKQFWIPELDNIQHFLGGMSGNALVGMGVLVALTTYWLASRHRA

ACSL1c_ENSTNIG00000010115      --------------------------------------------------
ACSL1a_ENSTNIG00000018054      VKQRVDFSRQSVELPGGEGIRRSVLVENDQLITHYYDDARTFYELFLRGL

ACSL1c_ENSTNIG00000010115      --------------------------------------------------
ACSL1a_ENSTNIG00000018054      RESNNGPCLGSRKLNHPYEWQSYQEVVADRAKHIGSALLNKGHSHTGDKF

ACSL1c_ENSTNIG00000010115      --------------------------------------------------
ACSL1a_ENSTNIG00000018054      IGIFSLNRPEWTISELACYTYSLVAVPLYDTLGREAIGYIIDKATISTLI

ACSL1c_ENSTNIG00000010115      --------------------------------------------------
```

```
ACSL1a_ENSTNIG00000018054      CDLPEKAWMVLDCINGKGKSVKRIVIMGPFQSELVERAEECDIEIISFED

ACSL1c_ENSTNIG00000010115      -----------------------CFGTVVSGDPKGAMLTHENIVSNCSAV
ACSL1a_ENSTNIG00000018054      FEALGQDTVMEPVPPAPEDLALVCFTSGTTGKPKGAMLTHGNIIANTAAF
                                                      ** : .:*.******** **::* :*.

ACSL1c_ENSTNIG00000010115      IKVTEVSCPFCSSDTHMSYLPLAHMFERIVQGVVLVHGARIGFFQGDIRS
ACSL1a_ENSTNIG00000018054      LKLTEKDCMLCVHDIHISYLPLAHMLERVIHGVVLVHGGRVGFFQGDIRL
                               :*:** .* :*  * *:********:**:::*******.*:********

ACSL1c_ENSTNIG00000010115      LSDDLCALKPTVFPVVPRLLNRMYDRIFGQANSTVKRWLLGFAFRRKEAE
ACSL1a_ENSTNIG00000018054      LMDDLQTLKPTVFPMVPRLLNRMCDKIFSQADTPLKKWLLRLAFSRKIAE
                               * *** :*******:******** *:**.**::.:*:*** :** ** **

ACSL1c_ENSTNIG00000010115      LRRGIMRRDSIWDRLIFRKVQASLGGRVRFMITGAAPISPAVLTFLRVAM
ACSL1a_ENSTNIG00000018054      LNQGVVRQDTIWDRLIFKKVQANTGGRVRMMITGAPPVCPKNLTYINITT
                               *.:*::*:*:*******:****. *****:*****.*:.*  **::.::

ACSL1c_ENSTNIG00000010115      GCQFFEGYGQTECTAGCTMTLAGDWTAGHVGPPLPCNSVKLVDVAEMNYL
ACSL1a_ENSTNIG00000018054      MLQLYEGYGQTECTAGCSMSLPGDWIAGAVGPPVPCNDIKLVDVAEMNYF
                                 *::************:*:*.*** ** ****:***.:**********:

ACSL1c_ENSTNIG00000010115      AANGEGEVCVKGPNVFQGYLHDPEKTAEAIDAHGWLHTGDIGKWLPNGTL
ACSL1a_ENSTNIG00000018054      AANGEGEVCAKGTNVFKGYLGDAEKTAEALDEDGWLHTGDIGKWLPNGTL
                               *********.**.***:*** *.******:* .*****************

ACSL1c_ENSTNIG00000010115      KIIDRKKHIFKLAQGEYIAPEKIENVYVRSSAVAQVYVHGDSLQAFLVAV
ACSL1a_ENSTNIG00000018054      KITDRKKNIFKMAQGEYIAPERIEMIYNRSEPVAQIFVHGDSLKACLVAI
                               ** ****:***:*********:** :* **..***::******:* ***:

ACSL1c_ENSTNIG00000010115      VVPDPDFLCGWAKKTLGLRGSYEDLCSKESVWVSVFERCVIVIEDACVVL
ACSL1a_ENSTNIG00000018054      VVPDSETLPDWIKK-KGIEGPPTGLCKNQDVKRAIQEDILRLGREAGLKS
                               ****.: * .* **  *:.*.  .**.::.*  :: *  : : .:* :

ACSL1c_ENSTNIG00000010115      CVQVKAIAIHPELFSVENGLLTPTLKAKRNEMRQFFRPQLDHLYASIKM
ACSL1a_ENSTNIG00000018054      FEQVKDITLHPEMFSIQNGLLTPTLKSKRVELRRYFRKQIDEMYAKIKR
                                 *** *::***:**::*********:** *:*::** *:*.:**.**
```
