# Supplementary material for: Diversity and history of the long-chain acyl-CoA synthetase (Acsl) gene family in vertebrates
Source: BMC Evol Biol. 2013 Dec 12;13:271. doi: 10.1186/1471-2148-13-271 (PMC3890633; doi:10.1186/1471-2148-13-271)
Supplement: Additional file 3 — NCBI accession numbers and Ensembl gene ID. [file 1471-2148-13-271-S3.pdf]

Additional file 3

NCBI accession numbers and ensemble gene ID numbers of the final set of ACSL sequences gathered after extensive database searches.

	ACSL1	ACSL3	ACSL4	ACSL5	ACSL6	ACSL2
Hs	NP_001986.2	NP_004448.2	NP_075266.1	NP_057318.2	NP_056071.2	na
Mm	NP_032007.2	NP_083093.2	NP_997508.1	NP_082252.1	NP_659072.3	na
Md	XP_001363547.1	XP_001365624.2	XP_001363499.1	XP_003339985.1	XM_001371426.1	na
Xt	NP_001006830.1	NP_001090679.1	XP_002938836.1	NP_001011069.1	NP_001072330.1	na
Ac	XP_003221679.1	XP_003218347.1	XP_003223418.1	XP_003223478.1	XP_003217452.1	na
Gg	NP_001012596.1	XP_422625.2	XP_420317.2	NP_001026408.1	ENSGALG0000000 6644	na
Oa	XP_003429626.1	ENSOANG0000001 3598	XP_001507836.2	XP_001513244.1	ENSOANG0000000 9215	na
Tr	ENSTRUG000000 17576	ENSTRUG0000000 9826	ENSTRUP0000000 6243	ENSTRUG0000000 7791	ENSTRUG0000000 4657	ENSTRUG0000000 4367
		ENSTRUG0000001 3537				
Ol	ENSORLG000000 18806	ENSORLG0000000 9215	ENSORLG0000000 8040	ENSORLG0000001 1808	ENSORLG0000000 1111	ENSORLG0000001 1037
	ENSORLG000000 08655	ENSORLG0000001 5909				
Ga	ENSGACG000000 17662	ENSGACG0000000 0252	ENSGACG0000001 8503	ENSGACG0000000 3579	ENSGACG0000002 0877	ENSGACG0000001 0837
	ENSGACG000000 18774	ENSGACG0000001 4028				
Tn	ENSTNIG000000 18054	ENSTNIG00000015 280	ENSTNIG0000001 5788	ENSTNIG0000001 4415	ENSTNIG0000001 6086	ENSTNIG0000001 0817
		ENSTNIG0000001 4309				
Dr	NP_001003569.1	ENSDARG0000003 2079	ENSDARG0000000 4078	ENSDARG0000007 5931	XP_001920939.3	ENSDARG0000007 8399
	CAX14650.1	ENSDARG0000001 4674	AAH91952.1			

Hs- *Homo sapiens*; **Mm-** *Mus musculus*; **Md-** *Monodelphis domestica*; **Xt-** *Xenopus tropicalis*; **Ac-** *Anolis carolinensis;* **Gg-** *Gallus gallus;* **Oa-** *Ornithorhynchus anatinus;* **Tr-** *Takifugu rubripes;* **Ol-** *Oryzias latipes;* **Ga-** *Gasterosteus aculeatus*; **Tn-** *Tetraodon nigroviridis;* **Dr-** *Danio rerio*
