# Supplementary figures and images for: Diversity and history of the long-chain acyl-CoA synthetase (Acsl) gene family in vertebrates
Source: BMC Evol Biol. 2013 Dec 12;13:271. doi: 10.1186/1471-2148-13-271 (PMC3890633; doi:10.1186/1471-2148-13-271)

**Synteny maps of Zebrafish ACSL4 3R duplicates**

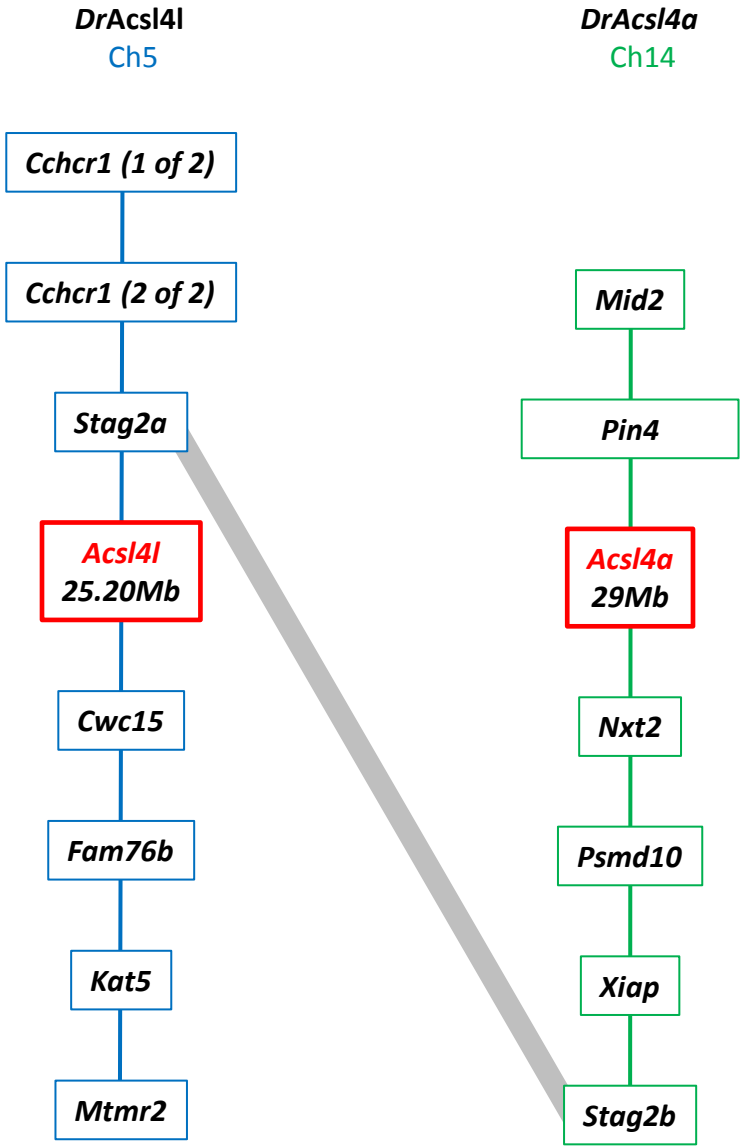

Supplement: Additional file 4 — Synteny maps of Zebrafish ACSL 3R duplicates. [file 1471-2148-13-271-S4.pdf]

***Xenopus tropicalis* *Acsl3* and *Acsl4* and corresponding genome location in human**

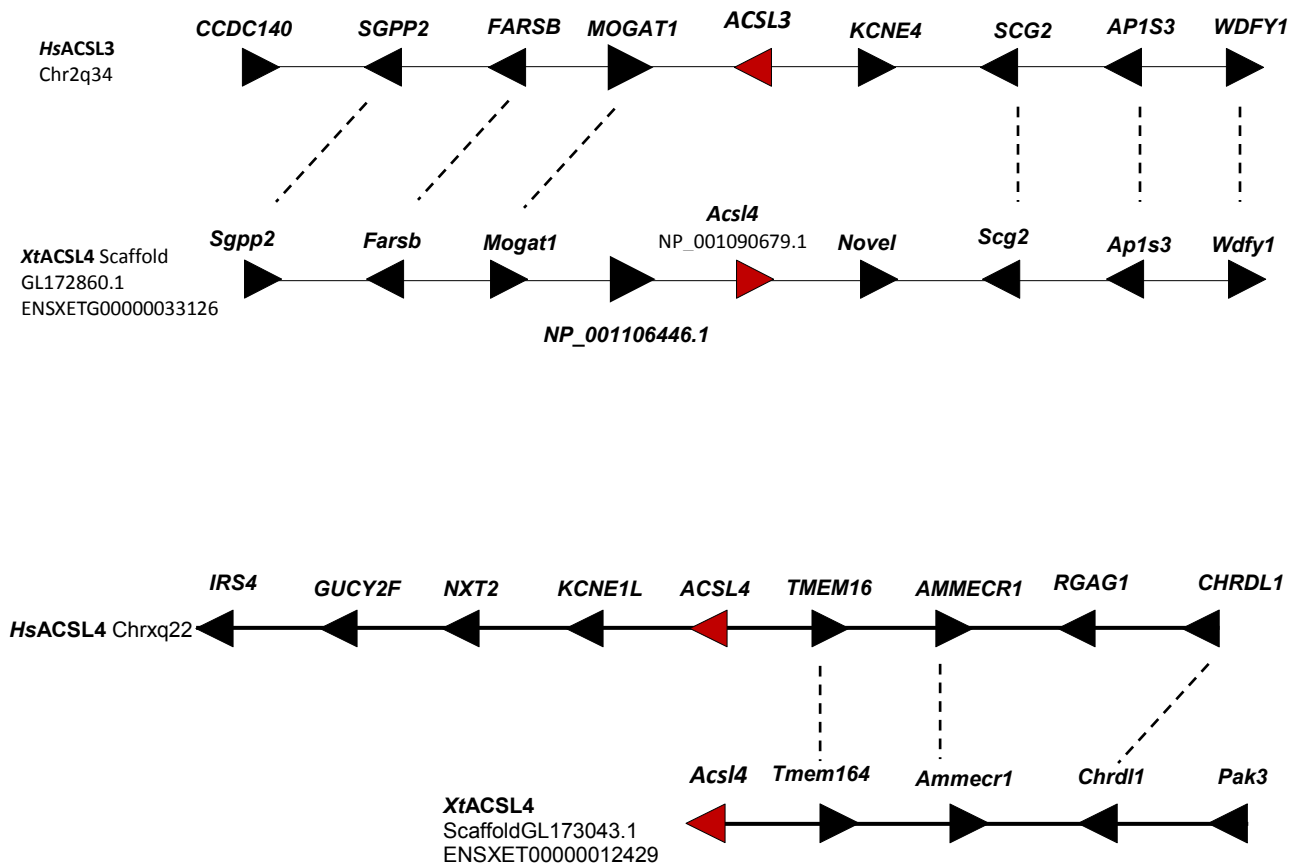

Supplement: Additional file 5 — Xenopus tropicalis Acsl4 and Acsl3 corresponding location in human. [file 1471-2148-13-271-S5.pdf]
